# Inferring topology from clustering coefficients in protein-protein interaction networks

**DOI:** 10.1186/1471-2105-7-519

**Published:** 2006-11-30

**Authors:** Caroline C Friedel, Ralf Zimmer

**Affiliations:** 1 LFE Bioinformatik, Institut für Informatik, Ludwig-Maximilians-Universität München, Amalienstraße 17, 80333 München, Germany

## Abstract

**Background:**

Although protein-protein interaction networks determined with high-throughput methods are incomplete, they are commonly used to infer the topology of the complete interactome. These partial networks often show a scale-free behavior with only a few proteins having many and the majority having only a few connections. Recently, the possibility was suggested that this scale-free nature may not actually reflect the topology of the complete interactome but could also be due to the error proneness and incompleteness of large-scale experiments.

**Results:**

In this paper, we investigate the effect of limited sampling on average clustering coefficients and how this can help to more confidently exclude possible topology models for the complete interactome. Both analytical and simulation results for different network topologies indicate that partial sampling alone lowers the clustering coefficient of all networks tremendously. Furthermore, we extend the original sampling model by also including spurious interactions via a preferential attachment process. Simulations of this extended model show that the effect of wrong interactions on clustering coefficients depends strongly on the skewness of the original topology and on the degree of randomness of clustering coefficients in the corresponding networks.

**Conclusion:**

Our findings suggest that the complete interactome is either highly skewed such as e.g. in scale-free networks or is at least highly clustered. Although the correct topology of the interactome may not be inferred beyond any reasonable doubt from the interaction networks available, a number of topologies can nevertheless be excluded with high confidence.

## Background

Since protein-protein interactions are of fundamental importance for all processes taking place in a cell, great efforts have been devoted to the systematic identification of protein interactions for a number of organisms. To generate large-scale protein interaction maps, two methods are commonly used: (i) yeast two-hybrid (Y2H) [1-7] and (ii) affinity purification followed by mass spectrometry (e.g. Co-immuno-precipitation (Co-IP) [8] or tandem affinity purification (TAP) [9-11]). Both of these methods are prone to spurious interactions (false positives) due to self-activators (Y2H), protein contaminants (affinity purification) or non-specific interactions. Based on expression data and information about paralogues, the fraction of correct high-throughput interactions has been estimated at 30–50% [12]. In addition to false positives, high-throughput experiments are characterized by a large fraction of false negatives, i.e. correct interactions that are missed in the experiment. Accordingly, only small overlaps can be observed between interaction maps for the same species but determined in different experiments and with different methods [13,14].

Despite the amount of false positives and false negatives associated with protein-protein interaction (PPI) networks determined in high-throughput experiments, they have nevertheless been thoroughly investigated in terms of network topology, stability and dynamics [15-20]. The topology of protein-protein interaction networks is in general described as scale-free, a topology common to many networks from various domains [21-23], although this claim has been questioned recently [24,25]. Scale-free networks are characterized by a power-law degree distribution in which the probability of a node having *k *interaction partners is proportional to *k*^-γ ^for some constant *γ*. Accordingly, the majority of nodes interact only with few other nodes, whereas a small fraction of nodes (so-called hubs) have connections to many other nodes in the network. As a consequence, scale-free networks are very tolerant to random deletion of nodes but vulnerable to a targeted attack against hubs. Indeed, lethality of protein knockouts appears to be correlated to the number of interaction partners of the protein [15].

All of these studies implicitly assume that the topology of the complete interactome can be inferred from observed PPI networks containing only a fraction of proteins and interactions. Recently, this assumption has been called into question [26,27]. Based on mathematical modeling, Stumpf et al. [26] showed that, unlike for random graph and exponential topologies, random sampling from scale-free networks has a distorting effect on the topology of sub-networks. Conversely, these results imply that the scale-free topology of the PPI networks is unlikely to result from random graphs or exponential networks by the random sampling approach postulated by Stumpf et al., which selects only a fraction of nodes and all edges between these nodes. Since such a random sampling procedure does not accurately reflect the impact of large-scale experimental methods, Han et al. [27] defined a different limited sampling procedure which emulates the effect of the Y2H approach. Based on simulations they argue that such a limited sampling can lead to an apparent scale-free topology in the sampled networks regardless of the original topology. They conclude that, while a scale-free topology appears to be more likely than the other models considered, these other topologies cannot be safely excluded based on the degree distribution alone given the currently available interaction data.

We proposed recently [28] that apart from the degree distribution and the related network statistics discussed by Han et al., other characteristics of the network might help to further assess the likelihood of different topology models and exclude at least some of them. One such characteristic is the average clustering coefficient, i.e. the "cliquishness" of the network. In this paper, we analyze the effect of the sampling procedure described by Han and co-workers on the clustering coefficient analytically in addition to simulations. Both our analytical and simulation results shown here suggest that random sampling with a limited coverage of proteins and interactions always leads to lower clustering in the resulting sub-network compared to the original network. As a consequence, in such a setting the clustering coefficients of protein interaction networks derived by Y2H can be considered as a lower bound on the clustering coefficients of the original networks and network topologies with significantly lower clustering coefficients than observed can be ruled out.

We furthermore extend the model of Han et al. by additionally adding spurious interactions to the sampled networks and analyze the effects of these false positive interactions both analytically and with simulations. Although false positive interactions can be viewed as another sampling artifact, their impact on the network might be different from limited sampling effects. Indeed, we observe that the average clustering coefficient of a network reacts differently to false positive interactions than to false negative interactions. In our model, interactions are added using a preferential attachment model [29] and, accordingly, false positive interactions alone can increase the skewness of the theoretical networks and, thus, their similarity to scale-free networks. Our findings show that although clustering coefficients of networks can be increased by wrong interactions for some network topologies, the degree to which they can be increased depends strongly on the degree of randomness of clustering coefficients and the degree distribution of the original topology. As a consequence, several topologies remain unlikely and can be excluded with high confidence.

## Results

### Modeling yeast-two hybrid experiments

A protein-protein interaction network can be described as an undirected graph *G = *(*V, E*) with a set of nodes *V *and a set of edges *E*. The nodes in *G *then correspond to interacting proteins and two nodes *u *and *v *are connected by an edge (*u, v*) if and only if they interact. Interactions may either be direct such as the physical interactions determined with Y2H or indirect via other proteins in the same complex as detected by affinity purification. Since these differences make it difficult to define a comprehensive model for both experimental methods, the sampling procedure described by Han et al. simulates only the Y2H approach to address direct interactions.

Although many topological properties can be analyzed, we concentrate on two of them, the degree distribution and the average clustering coefficient. The degree *k*_*v *_of a node *v *is the number of its interactions and the average degree of all nodes in the graph is denoted by k¯
 MathType@MTEF@5@5@+=feaafiart1ev1aaatCvAUfKttLearuWrP9MDH5MBPbIqV92AaeXatLxBI9gBaebbnrfifHhDYfgasaacH8akY=wiFfYdH8Gipec8Eeeu0xXdbba9frFj0=OqFfea0dXdd9vqai=hGuQ8kuc9pgc9s8qqaq=dirpe0xb9q8qiLsFr0=vr0=vr0dc8meaabaqaciaacaGaaeqabaqabeGadaaakeaacuWGRbWAgaqeaaaa@2E23@. Thus, the degree distribution describes the probability of a node *v *having degree *k*:

P(k)=|{v∈V|kv=k}||V|.     (1)
 MathType@MTEF@5@5@+=feaafiart1ev1aaatCvAUfKttLearuWrP9MDH5MBPbIqV92AaeXatLxBI9gBaebbnrfifHhDYfgasaacH8akY=wiFfYdH8Gipec8Eeeu0xXdbba9frFj0=OqFfea0dXdd9vqai=hGuQ8kuc9pgc9s8qqaq=dirpe0xb9q8qiLsFr0=vr0=vr0dc8meaabaqaciaacaGaaeqabaqabeGadaaakeaacqWGqbaucqGGOaakcqWGRbWAcqGGPaqkcqGH9aqpdaWcaaqaaiabcYha8jabcUha7jabdAha2jabgIGiolabdAfawjabcYha8jabdUgaRnaaBaaaleaacqWG2bGDaeqaaOGaeyypa0Jaem4AaSMaeiyFa0NaeiiFaWhabaGaeiiFaWNaemOvayLaeiiFaWhaaiabc6caUiaaxMaacaWLjaWaaeWaaeaacqaIXaqmaiaawIcacaGLPaaaaaa@4BEF@

As we will see later, an important characteristic of the degree distribution is its asymmetry, i.e. its skewness. Although there exist several alternative definitions of skewness, the one most commonly used is

skewness=∑v∈V(kv−k¯)3(|V|−1)s3     (2)
 MathType@MTEF@5@5@+=feaafiart1ev1aaatCvAUfKttLearuWrP9MDH5MBPbIqV92AaeXatLxBI9gBaebbnrfifHhDYfgasaacH8akY=wiFfYdH8Gipec8Eeeu0xXdbba9frFj0=OqFfea0dXdd9vqai=hGuQ8kuc9pgc9s8qqaq=dirpe0xb9q8qiLsFr0=vr0=vr0dc8meaabaqaciaacaGaaeqabaqabeGadaaakeaacqWGZbWCcqWGRbWAcqWGLbqzcqWG3bWDcqWGUbGBcqWGLbqzcqWGZbWCcqWGZbWCcqGH9aqpdaWcaaqaamaaqababaGaeiikaGIaem4AaS2aaSbaaSqaaiabdAha2bqabaGccqGHsislcuWGRbWAgaqeaiabcMcaPmaaCaaaleqabaGaeG4mamdaaaqaaiabdAha2jabgIGiolabdAfawbqab0GaeyyeIuoaaOqaaiabcIcaOiabcYha8jabdAfawjabcYha8jabgkHiTiabigdaXiabcMcaPiabdohaZnaaCaaaleqabaGaeG4mamdaaaaakiaaxMaacaWLjaWaaeWaaeaacqaIYaGmaiaawIcacaGLPaaaaaa@555E@

where *s *is the sample standard deviation of the degree distribution. For symmetric distributions the skewness is close to zero whereas for left-tailed distributions it is negative and for right-tailed distribution, such as e.g. power-law distributions, it is positive.

The clustering coefficient quantifies the probability that two vertices which are connected to the same node are also connected. Accordingly, the clustering coefficient *C*_*v *_of a node *v *in a network is defined as [30]

Cv=P((u,w)∈E|(u,v)∈E∧(v,w)∈E)=P((u,w)∈E∧(u,v)∈E∧(v,w)∈E)P((u,v)∈E∧(v,w)∈E)=:P(∇∈E)P(∨∈E).     (3)
 MathType@MTEF@5@5@+=feaafiart1ev1aaatCvAUfKttLearuWrP9MDH5MBPbIqV92AaeXatLxBI9gBaebbnrfifHhDYfgasaacH8akY=wiFfYdH8Gipec8Eeeu0xXdbba9frFj0=OqFfea0dXdd9vqai=hGuQ8kuc9pgc9s8qqaq=dirpe0xb9q8qiLsFr0=vr0=vr0dc8meaabaqaciaacaGaaeqabaqabeGadaaakeaafaqadeWabaaabaGaem4qam0aaSbaaSqaaiabdAha2bqabaGccqGH9aqpcqWGqbaucqGGOaakcqGGOaakcqWG1bqDcqGGSaalcqWG3bWDcqGGPaqkcqGHiiIZcqWGfbqrcqGG8baFcqGGOaakcqWG1bqDcqGGSaalcqWG2bGDcqGGPaqkcqGHiiIZcqWGfbqrcqGHNis2cqGGOaakcqWG2bGDcqGGSaalcqWG3bWDcqGGPaqkcqGHiiIZcqWGfbqrcqGGPaqkaeaacqGH9aqpdaWcaaqaaiabdcfaqjabcIcaOiabcIcaOiabdwha1jabcYcaSiabdEha3jabcMcaPiabgIGiolabdweafjabgEIizlabcIcaOiabdwha1jabcYcaSiabdAha2jabcMcaPiabgIGiolabdweafjabgEIizlabcIcaOiabdAha2jabcYcaSiabdEha3jabcMcaPiabgIGiolabdweafjabcMcaPaqaaiabdcfaqjabcIcaOiabcIcaOiabdwha1jabcYcaSiabdAha2jabcMcaPiabgIGiolabdweafjabgEIizlabcIcaOiabdAha2jabcYcaSiabdEha3jabcMcaPiabgIGiolabdweafjabcMcaPaaaaeaacqGH9aqpcqGG6aGodaWcaaqaaiabdcfaqjabcIcaOiabgEGirlabgIGiolabdweafjabcMcaPaqaaiabdcfaqjabcIcaOiabgIIiAlabgIGiolabdweafjabcMcaPaaacqGGUaGlaaGaaCzcaiaaxMaadaqadaqaaiabiodaZaGaayjkaiaawMcaaaaa@97C5@

Since the clustering coefficient is only defined for nodes with at least two neighbors, the clustering coefficient *C *of the complete network is defined as the average clustering coefficient of all nodes with degree at least 2.

**Figure 1:**
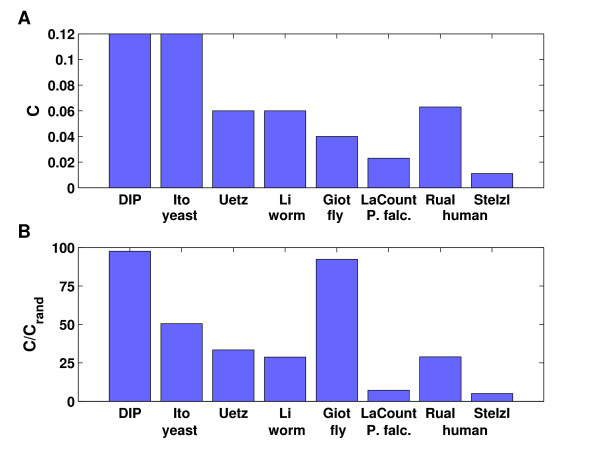
**Clustering coefficients in large-scale Y2H interaction networks**. Clustering coefficients (**A**) and the ratio to clustering coefficients of random graphs [31] having the same size (**B**) are shown for the following interaction networks: yeast interactions from DIP [33] and the Y2H studies by Ito et al. [2] and Uetz et al. [1]; C. elegans interactions by Li et al. [3]; drosophila interactions by Giot et al. [4]; P. falciparum interactions by LaCount et al. [5]; and human interactions from the studies of Rual et al. [6] and Stelzl et al. [7]. Only high confidence interactions were considered for the Ito, Li and Giot data set and self-edges were ignored for the calculation of clustering coefficients.

Average clustering coefficients of networks are often compared against the clustering coefficients of random graphs [31] containing the same number of nodes and edges. The expected clustering coefficient of such a random graph is 2|E||V|(|V|−1)
 MathType@MTEF@5@5@+=feaafiart1ev1aaatCvAUfKttLearuWrP9MDH5MBPbIqV92AaeXatLxBI9gBaebbnrfifHhDYfgasaacH8akY=wiFfYdH8Gipec8Eeeu0xXdbba9frFj0=OqFfea0dXdd9vqai=hGuQ8kuc9pgc9s8qqaq=dirpe0xb9q8qiLsFr0=vr0=vr0dc8meaabaqaciaacaGaaeqabaqabeGadaaakeaadaWcaaqaaiabikdaYiabcYha8jabdweafjabcYha8bqaaiabcYha8jabdAfawjabcYha8jabcIcaOiabcYha8jabdAfawjabcYha8jabgkHiTiabigdaXiabcMcaPaaaaaa@3DBA@. Since most networks show a degree distribution different from random graphs, it is also useful to compare these networks against random networks with the same degree distribution. Such networks can be easily obtained by randomly rewiring edges many times such that the degree distribution is preserved [32]. Here, rewiring consists in randomly deleting two edges (*u, v*) and (*w, x*) and replacing them by two edges (*u, x*) and (*w, v*). We say that a network is clustered randomly if after rewiring approximately the same clustering coefficients are observed. Consequently, a network can be clustered randomly, less than randomly or more than randomly. We will see examples for all three cases later on. Figure 1A shows the average clustering coefficients for a number of high-throughput Y2H data sets. Here, only high-confidence interactions were considered for the data sets of Ito et al. [2], Li et al. [3] and Giot et al. [4]. For comparison purposes, the same characteristics are given for the yeast protein-protein interaction network from DIP [33] which contains high-throughput data as well as interactions determined with other experimental methods. Although the clustering coefficients of some of the partial networks appear to be rather small, they are in most cases at least one order of magnitude higher than clustering coefficients of random graphs with the same number of nodes and edges (see Figure 1B).

#### Missing interactions

The sampling procedure described by Han et al. [27] emulates the effect of the Y2H method under the assumption that interactions may be missed in the process but no wrong interactions are obtained. It is determined uniquely by two parameters: bait coverage (denoted by *β*) and edge coverage (denoted by *ε*). Bait coverage specifies the selective effect of choosing only a fraction of the proteome as baits in a large-scale yeast two-hybrid experiment, whereas edge coverage determines the fraction of true interactions which can actually be resolved for a bait. Accordingly, a network is sampled from the original network as follows. A fraction *β *of nodes is selected as baits and then for each bait a fraction *ε *of its interactions. Edges connecting two baits are selected with higher probability 2*ε *- *ε*^2 ^= *ε*(2 - *ε*). The sampled network then contains the bait nodes as well as non-bait nodes which are connected to a bait via a sampled edge. In the following, the latter ones are referred to as preys. The resulting network is referred to as G^1 ^= (*V*^1^, *E*^1^) and the set of baits is called *B*. The resulting degree of a node *v *and its clustering coefficient are consequently referred to as kv1
 MathType@MTEF@5@5@+=feaafiart1ev1aaatCvAUfKttLearuWrP9MDH5MBPbIqV92AaeXatLxBI9gBaebbnrfifHhDYfgasaacH8akY=wiFfYdH8Gipec8Eeeu0xXdbba9frFj0=OqFfea0dXdd9vqai=hGuQ8kuc9pgc9s8qqaq=dirpe0xb9q8qiLsFr0=vr0=vr0dc8meaabaqaciaacaGaaeqabaqabeGadaaakeaacqWGRbWAdaqhaaWcbaGaemODayhabaGaeGymaedaaaaa@309D@ and Cv1
 MathType@MTEF@5@5@+=feaafiart1ev1aaatCvAUfKttLearuWrP9MDH5MBPbIqV92AaeXatLxBI9gBaebbnrfifHhDYfgasaacH8akY=wiFfYdH8Gipec8Eeeu0xXdbba9frFj0=OqFfea0dXdd9vqai=hGuQ8kuc9pgc9s8qqaq=dirpe0xb9q8qiLsFr0=vr0=vr0dc8meaabaqaciaacaGaaeqabaqabeGadaaakeaacqWGdbWqdaqhaaWcbaGaemODayhabaGaeGymaedaaaaa@304D@. The average degree of the network and the average clustering coefficient are denoted by k¯
 MathType@MTEF@5@5@+=feaafiart1ev1aaatCvAUfKttLearuWrP9MDH5MBPbIqV92AaeXatLxBI9gBaebbnrfifHhDYfgasaacH8akY=wiFfYdH8Gipec8Eeeu0xXdbba9frFj0=OqFfea0dXdd9vqai=hGuQ8kuc9pgc9s8qqaq=dirpe0xb9q8qiLsFr0=vr0=vr0dc8meaabaqaciaacaGaaeqabaqabeGadaaakeaacuWGRbWAgaqeaaaa@2E23@^1 ^and *C*^1^.

#### Spurious interactions

Since false positive interactions may affect both the degree distribution and the clustering coefficient, we extended the simple sampling model to include also wrong interactions. For this purpose, the sampling procedure is modified in the following way. In the sampling step all nodes of the original network are retained but only interactions which involve at least one bait. False positive interactions are then added in a second step. For each bait *v *we add an interaction to any other node *u *with a specific probability *ω*(*v, u*) and the resulting network is denoted as G^2 ^= (*V*^2^, *E*^2^).

The probability *ω*(*v, u*) can be defined in different ways. In the first case, the probability of adding an edge between *v *and *u *depends neither on the degree of *v *or *u*, i.e. is constant for all pairs of nodes. In a similar way, Erdős and Rényi random graphs [31] are created and thus this process is denoted as random attachment. In the second case *ω*(*v, u*) does only depend on the degree of the bait *v *but is constant for all its possible neighbors *u*. We denote this behavior as semi-preferential attachment, since new edges will be attached preferentially to baits with high degree. The last possible scenario involves preferential attachment for both *v *and *u*.

Since preferential attachment is most likely to change the degree distribution towards a power-law distribution [29], our model is based on such a scenario. For this purpose, we use an adaption of the method described by Chung and Lu [34] for creating random graphs with a given degree distribution. Accordingly, *ω*(*v, u*) is defined as

ω(v,u)=θ(kv+ι)(ku+ι)∑w∈V(kw+ι).     (4)
 MathType@MTEF@5@5@+=feaafiart1ev1aaatCvAUfKttLearuWrP9MDH5MBPbIqV92AaeXatLxBI9gBaebbnrfifHhDYfgasaacH8akY=wiFfYdH8Gipec8Eeeu0xXdbba9frFj0=OqFfea0dXdd9vqai=hGuQ8kuc9pgc9s8qqaq=dirpe0xb9q8qiLsFr0=vr0=vr0dc8meaabaqaciaacaGaaeqabaqabeGadaaakeaaiiGacqWFjpWDcqGGOaakcqWG2bGDcqGGSaalcqWG1bqDcqGGPaqkcqGH9aqpcqWF4oqCdaWcaaqaaiabcIcaOiabdUgaRnaaBaaaleaacqWG2bGDaeqaaOGaey4kaSIae8xUdKMaeiykaKIaeiikaGIaem4AaS2aaSbaaSqaaiabdwha1bqabaGccqGHRaWkcqWF5oqAcqGGPaqkaeaadaaeqaqaaiabcIcaOiabdUgaRnaaBaaaleaacqWG3bWDaeqaaOGaey4kaSIae8xUdKMaeiykaKcaleaacqWG3bWDcqGHiiIZcqWGwbGvaeqaniabggHiLdaaaOGaeiOla4IaaCzcaiaaxMaadaqadaqaaiabisda0aGaayjkaiaawMcaaaaa@575D@

Note that *k*_*v *_denotes the degree of node *v *in the original network. Thus, the number of wrong interactions a protein obtains depends on the number of true interactions it forms. This is based on the assumption that highly interactive proteins are more prone to spurious interactions than proteins which form only a few but very specific interactions. The parameter *θ *controls the false positive rate, whereas *ι *is used as a pseudo-count to guarantee that singular nodes, i.e. nodes with degree zero, can also obtain wrong interactions. We have that 0 ≤ *ι *< ∞ and the larger *ι *the smaller is the influence of the actual degree values of *v *and *u *on the probability *ω*(*v, u*). For our purposes, *ι *was set to 1.

### Analytical results

In the following, theoretical derivations are given which describe the influence of the complete model on the clustering coefficient of networks. For simplification, we address the effect of limited sampling, i.e. missing interactions, and false positives, i.e. spurious interactions, separately from each other.

#### Missing interactions

In this section, we analyze the effect of limited sampling on the clustering coefficient of a node and the complete network. We show that both limited bait coverage and limited edge coverage leads to a reduction in clustering coefficients and therefore that limited sampling as a whole lowers the clustering coefficient. Again, the clustering coefficient of a node *v *after sampling can be formulated as a conditional probability:

Cv1=P(∇∈E1)P(∨∈E1)=P(∇∈E1|∇∈E)P(∨∈E1|∨∈E)P(∇∈E)P(∨∈E)=P(∇∈E1|∇∈E)P(∨∈E1|∨∈E)Cv.     (5)
 MathType@MTEF@5@5@+=feaafiart1ev1aaatCvAUfKttLearuWrP9MDH5MBPbIqV92AaeXatLxBI9gBaebbnrfifHhDYfgasaacH8akY=wiFfYdH8Gipec8Eeeu0xXdbba9frFj0=OqFfea0dXdd9vqai=hGuQ8kuc9pgc9s8qqaq=dirpe0xb9q8qiLsFr0=vr0=vr0dc8meaabaqaciaacaGaaeqabaqabeGadaaakeaafaqadeGabaaabaGaem4qam0aa0baaSqaaiabdAha2bqaaiabigdaXaaakiabg2da9maalaaabaGaemiuaaLaeiikaGIaey4bIeTaeyicI4Saemyrau0aaWbaaSqabeaacqaIXaqmaaGccqGGPaqkaeaacqWGqbaucqGGOaakcqGHOiI2cqGHiiIZcqWGfbqrdaahaaWcbeqaaiabigdaXaaakiabcMcaPaaacqGH9aqpdaWcaaqaaiabdcfaqjabcIcaOiabgEGirlabgIGiolabdweafnaaCaaaleqabaGaeGymaedaaOGaeiiFaWNaey4bIeTaeyicI4SaemyrauKaeiykaKcabaGaemiuaaLaeiikaGIaeyikIOTaeyicI4Saemyrau0aaWbaaSqabeaacqaIXaqmaaGccqGG8baFcqGHOiI2cqGHiiIZcqWGfbqrcqGGPaqkaaWaaSaaaeaacqWGqbaucqGGOaakcqGHhis0cqGHiiIZcqWGfbqrcqGGPaqkaeaacqWGqbaucqGGOaakcqGHOiI2cqGHiiIZcqWGfbqrcqGGPaqkaaaabaGaeyypa0ZaaSaaaeaacqWGqbaucqGGOaakcqGHhis0cqGHiiIZcqWGfbqrdaahaaWcbeqaaiabigdaXaaakiabcYha8jabgEGirlabgIGiolabdweafjabcMcaPaqaaiabdcfaqjabcIcaOiabgIIiAlabgIGiolabdweafnaaCaaaleqabaGaeGymaedaaOGaeiiFaWNaeyikIOTaeyicI4SaemyrauKaeiykaKcaaiabdoeadnaaBaaaleaacqWG2bGDaeqaaOGaeiOla4caaiaaxMaacaWLjaWaaeWaaeaacqaI1aqnaiaawIcacaGLPaaaaaa@9135@

Thus, the clustering coefficient of node *v *depends on its original clustering coefficient and the probabilities *P*(∇ ∈ *E*^1^|∇ ∈ *E*) and *P*(∨ ∈ *E*^1^|∨ ∈ *E*).

**Figure 2:**
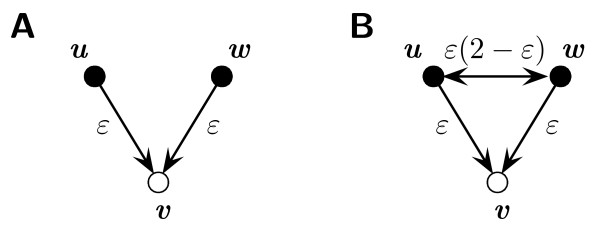
**Effect of sampling on preys**. This Figure illustrates the probabilities for selecting edges in the limited sampling step if the node *v *considered is a prey. Here, baits are indicated by black nodes and preys by white notes. The arrows at the end of edges indicate the bait and prey relationship for this edge and edges are directed from bait to prey. Accordingly, edges between baits show arrows at both ends.

To examine the full impact of sampling on the clustering coefficient of a node *v*, we have to differentiate between baits and preys. First, let *v *be a prey. In this case, both edges (*u, v*) and (*v, w*) can only be conserved if both *u *and *w *are chosen as baits. If at least on of them is not a bait, the corresponding edge to *v *is always missed. However, if both nodes are baits, the two edges connecting them to *v *are each selected with probability *ε *(see Figure 2A), since they connect a bait to a prey. Furthermore, these probabilities are then independent of each other and the joint probability that both edges are kept can be expressed by the product of the individual probabilities. As a consequence, we have that

*P*(∨ ∈ *E*^1^|∨ ∈ *E*) = *β*^2^*ε*^2^.     (6)

If *u *and *w *are connected in *G*, the corresponding edge again can only be selected if both nodes are baits. If this is true, the probability that this edge is conserved is then *ε*(2 - *ε*), since it connects two baits.

Accordingly, we have that

*P*(∇ ∈ *E*^1^|∇ ∈ *E*) = *β*^2^*ε*(2 - *ε*)*ε*^2 ^    (7)

and

Cv1
 MathType@MTEF@5@5@+=feaafiart1ev1aaatCvAUfKttLearuWrP9MDH5MBPbIqV92AaeXatLxBI9gBaebbnrfifHhDYfgasaacH8akY=wiFfYdH8Gipec8Eeeu0xXdbba9frFj0=OqFfea0dXdd9vqai=hGuQ8kuc9pgc9s8qqaq=dirpe0xb9q8qiLsFr0=vr0=vr0dc8meaabaqaciaacaGaaeqabaqabeGadaaakeaacqWGdbWqdaqhaaWcbaGaemODayhabaGaeGymaedaaaaa@304D@ = *ε*(2 - *ε*)*C*_*v *_≤ *C*_*v*_.     (8)

We thus observe that the clustering coefficient of a bait is only affected by limited edge coverage. If *ε *= 1, the expected clustering coefficient after sampling is approximately the same as before sampling regardless of the value of bait coverage.

**Figure 3:**
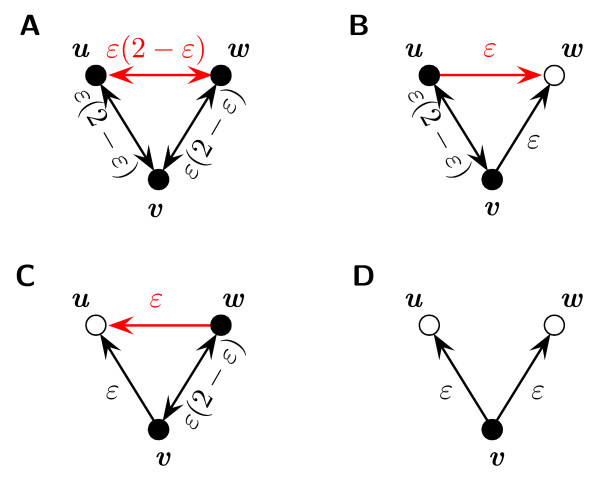
**Effect of sampling on baits**. Here, the probabilities for selecting edges are shown for the case that *v *is a bait. The notation is the same as in Figure 2. For each possible bait-prey combination of *u *and *w*, the probabilities are shown separately. The edge completing the triangle and the corresponding probabilities for selecting this edge are shown in red.

Second, let now *v *be a bait. In this case, the edges (*u, v*) and (*v, w*) can be conserved no matter if either of the nodes *u *and *w *is a bait or a prey. If both nodes are baits (see Figure 3A), each edge is selected with probability *ε*(2 - *ε*). If only one of them is a bait (Figure 3B and 3C), one edge is selected with probability *ε *and the other one with probability *ε*(2 - *ε*). If both are preys (Figure 3D), both edges are only selected with probability *ε*. Thus, we observe that

*P*(∨ ∈ *E*^1^|∨ ∈ *E*) = *β*^2^*ε*^2^(2 - *ε*)^2 ^+ 2*β*(1 - *β*)*ε*^2^(2 - *ε*) + (1 - *β*)^2^*ε*^2^.     (9)

On the other hand, a triangle between *u, v *and *w *can only be conserved if at least one of the two nodes *u *or *w *is also a bait. The probabilities for selecting edges (*u, v*) or (*v, w*) are in these cases the same as above. The third edge (*u, w*) is then selected with probability *ε*(2 - *ε*) if both nodes are baits and with probability *ε *if only one of the two nodes is a bait (see also Figure 3). Accordingly, we have that

*P*(∇ ∈ *E*^1^|∇ ∈ *E*) = *β*^2^*ε*^3^(2 - *ε*)^3 ^+ 2*β*(1 - *β*)*ε*^3^(2 - *ε*).     (10)

By inserting equations (9) and (10) into (5) we obtain that

Cv1
 MathType@MTEF@5@5@+=feaafiart1ev1aaatCvAUfKttLearuWrP9MDH5MBPbIqV92AaeXatLxBI9gBaebbnrfifHhDYfgasaacH8akY=wiFfYdH8Gipec8Eeeu0xXdbba9frFj0=OqFfea0dXdd9vqai=hGuQ8kuc9pgc9s8qqaq=dirpe0xb9q8qiLsFr0=vr0=vr0dc8meaabaqaciaacaGaaeqabaqabeGadaaakeaacqWGdbWqdaqhaaWcbaGaemODayhabaGaeGymaedaaaaa@304D@ = *ε*(2 - *ε*)*λC*_*v *_    (11)

with

λ:=β2(2−ε)2+2β(1−β)β2(2−ε)2+2β(1−β)(2−ε)+(1−β)2.     (12)
 MathType@MTEF@5@5@+=feaafiart1ev1aaatCvAUfKttLearuWrP9MDH5MBPbIqV92AaeXatLxBI9gBaebbnrfifHhDYfgasaacH8akY=wiFfYdH8Gipec8Eeeu0xXdbba9frFj0=OqFfea0dXdd9vqai=hGuQ8kuc9pgc9s8qqaq=dirpe0xb9q8qiLsFr0=vr0=vr0dc8meaabaqaciaacaGaaeqabaqabeGadaaakeaaiiGacqWF7oaBcqGG6aGocqGH9aqpdaWcaaqaaiab=j7aInaaCaaaleqabaGaeGOmaidaaOGaeiikaGIaeGOmaiJaeyOeI0Iae8xTduMaeiykaKYaaWbaaSqabeaacqaIYaGmaaGccqGHRaWkcqaIYaGmcqWFYoGycqGGOaakcqaIXaqmcqGHsislcqWFYoGycqGGPaqkaeaacqWFYoGydaahaaWcbeqaaiabikdaYaaakiabcIcaOiabikdaYiabgkHiTiab=v7aLjabcMcaPmaaCaaaleqabaGaeGOmaidaaOGaey4kaSIaeGOmaiJae8NSdiMaeiikaGIaeGymaeJaeyOeI0Iae8NSdiMaeiykaKIaeiikaGIaeGOmaiJaeyOeI0Iae8xTduMaeiykaKIaey4kaSIaeiikaGIaeGymaeJaeyOeI0Iae8NSdiMaeiykaKYaaWbaaSqabeaacqaIYaGmaaaaaOGaeiOla4IaaCzcaiaaxMaadaqadaqaaiabigdaXiabikdaYaGaayjkaiaawMcaaaaa@65ED@

It is easy to see that *λ *≤ 1 since

*β*^2^(2 - *ε*)^2 ^+ 2*β*(1 - *β*)

≤ *β*^2^(2 - *ε*)^2 ^+ 2*β*(1 - *β*)(2 - *ε*)

≤ *β*^2^(2 - *ε*)^2 ^+ 2*β*(1 - *β*)(2 - *ε*) + (1 - *β*)^2^.     (13)

As a consequence we have that Cv1
 MathType@MTEF@5@5@+=feaafiart1ev1aaatCvAUfKttLearuWrP9MDH5MBPbIqV92AaeXatLxBI9gBaebbnrfifHhDYfgasaacH8akY=wiFfYdH8Gipec8Eeeu0xXdbba9frFj0=OqFfea0dXdd9vqai=hGuQ8kuc9pgc9s8qqaq=dirpe0xb9q8qiLsFr0=vr0=vr0dc8meaabaqaciaacaGaaeqabaqabeGadaaakeaacqWGdbWqdaqhaaWcbaGaemODayhabaGaeGymaedaaaaa@304D@ ≤ *ε*(2 - *ε*)*C*_*v *_and in particular that Cv1
 MathType@MTEF@5@5@+=feaafiart1ev1aaatCvAUfKttLearuWrP9MDH5MBPbIqV92AaeXatLxBI9gBaebbnrfifHhDYfgasaacH8akY=wiFfYdH8Gipec8Eeeu0xXdbba9frFj0=OqFfea0dXdd9vqai=hGuQ8kuc9pgc9s8qqaq=dirpe0xb9q8qiLsFr0=vr0=vr0dc8meaabaqaciaacaGaaeqabaqabeGadaaakeaacqWGdbWqdaqhaaWcbaGaemODayhabaGaeGymaedaaaaa@304D@ <*ε*(2 - *ε*)*C*_*v *_if either *β *< 1 or *ε *< 1. This shows that both limited bait coverage as well as limited edge coverage lower the clustering coefficients of baits. Since *G*^1 ^contains at least one bait, we can conclude that Cv1
 MathType@MTEF@5@5@+=feaafiart1ev1aaatCvAUfKttLearuWrP9MDH5MBPbIqV92AaeXatLxBI9gBaebbnrfifHhDYfgasaacH8akY=wiFfYdH8Gipec8Eeeu0xXdbba9frFj0=OqFfea0dXdd9vqai=hGuQ8kuc9pgc9s8qqaq=dirpe0xb9q8qiLsFr0=vr0=vr0dc8meaabaqaciaacaGaaeqabaqabeGadaaakeaacqWGdbWqdaqhaaWcbaGaemODayhabaGaeGymaedaaaaa@304D@ <*C*_*v *_if bait or edge coverage is limited.

The sampling procedure described by Han et al. [27] corresponds to an experimental setting in which only a small set of proteins is chosen as baits and then subsequently screened against a much larger set of preys. This set-up is often used when due to a large genome size an exhaustive search for all possible protein pairs is infeasible [35]. An alternative approach consists in doing such exhaustive pairwise screens only for a subset of the proteome (see e.g. the human interaction network by Rual et al. [6]). We can easily reduce this scenario to the one considered here if we set *G *as the subgraph of the original network containing only the bait nodes and all edges between these nodes. Thus, we only need to consider the additional effect of this reduction. It can be shown that clustering coefficients of nodes selected for the screen remain approximately constant and, hence, that the average clustering coefficient of the subgraph *G *is approximately the same as in the original network. As a consequence, the matrix screen is reduced to a simple case of our model with *β *= 1 and we have that *C*^1 ^= *ε*(2 - *ε*)*C *with *C *the original clustering coefficient of the complete network.

#### Spurious interactions

In the first step discussed above, the possibility of additional spurious interactions is ignored and accordingly the probability is zero that edges which have not been part of the original network occur in the sampled network. However, since exactly this happens in the second step, we have that

*P*(◇ ∈ *E*^2^) = *P*(◇ ∈ *E*^2^|◇ ∈ *E*^1^)*P*(◇ ∈ *E*^1^) + *P*(◇ ∈ *E*^2^|◇ ∉ *E*^1^)*P*(◇ ∉ *E*^1^).     (14)

with ◇ ∈ {∨, ∇}.

In general, the resulting clustering coefficient is difficult to determine theoretically since *C*^2 ^cannot be given relative to *C*^1 ^as in the previous step. Therefore, we determine an approximation for the clustering coefficient only for the simple case that *β *= 1 and *ε *= 0, i.e. all proteins are selected as baits and none of the true edges are found. Thus, we see that

Cv2=P(∇∈E2|∇∈E1)⋅0+P(∇∈E2|∇∉E1)⋅1P(∨∈E2|∨∈E1)⋅0+P(∨∈E2|∨∉E1)⋅1=P(∇∈E2|∇∉E1)P(∨∈E2|∨∉E1).     (15)
 MathType@MTEF@5@5@+=feaafiart1ev1aaatCvAUfKttLearuWrP9MDH5MBPbIqV92AaeXatLxBI9gBaebbnrfifHhDYfgasaacH8akY=wiFfYdH8Gipec8Eeeu0xXdbba9frFj0=OqFfea0dXdd9vqai=hGuQ8kuc9pgc9s8qqaq=dirpe0xb9q8qiLsFr0=vr0=vr0dc8meaabaqaciaacaGaaeqabaqabeGadaaakeaacqWGdbWqdaqhaaWcbaGaemODayhabaGaeGOmaidaaOGaeyypa0ZaaSaaaeaacqWGqbaucqGGOaakcqGHhis0cqGHiiIZcqWGfbqrdaahaaWcbeqaaiabikdaYaaakiabcYha8jabgEGirlabgIGiolabdweafnaaCaaaleqabaGaeGymaedaaOGaeiykaKIaeyyXICTaeGimaaJaey4kaSIaemiuaaLaeiikaGIaey4bIeTaeyicI4Saemyrau0aaWbaaSqabeaacqaIYaGmaaGccqGG8baFcqGHhis0cqGHjiYZcqWGfbqrdaahaaWcbeqaaiabigdaXaaakiabcMcaPiabgwSixlabigdaXaqaaiabdcfaqjabcIcaOiabgIIiAlabgIGiolabdweafnaaCaaaleqabaGaeGOmaidaaOGaeiiFaWNaeyikIOTaeyicI4Saemyrau0aaWbaaSqabeaacqaIXaqmaaGccqGGPaqkcqGHflY1cqaIWaamcqGHRaWkcqWGqbaucqGGOaakcqGHOiI2cqGHiiIZcqWGfbqrdaahaaWcbeqaaiabikdaYaaakiabcYha8jabgIIiAlabgMGiplabdweafnaaCaaaleqabaGaeGymaedaaOGaeiykaKIaeyyXICTaeGymaedaaiabg2da9maalaaabaGaemiuaaLaeiikaGIaey4bIeTaeyicI4Saemyrau0aaWbaaSqabeaacqaIYaGmaaGccqGG8baFcqGHhis0cqGHjiYZcqWGfbqrdaahaaWcbeqaaiabigdaXaaakiabcMcaPaqaaiabdcfaqjabcIcaOiabgIIiAlabgIGiolabdweafnaaCaaaleqabaGaeGOmaidaaOGaeiiFaWNaeyikIOTaeyycI8Saemyrau0aaWbaaSqabeaacqaIXaqmaaGccqGGPaqkaaGaeiOla4IaaCzcaiaaxMaadaqadaqaaiabigdaXiabiwda1aGaayjkaiaawMcaaaaa@A128@

We furthermore assume that the probability that two nodes are connected is independent of the probability that any other two nodes are connected. In general, this is not the case for the preferential attachment model since the assumption holds only if all possible false positive edges are equally likely and, thus, if all nodes have approximately the same degree. Nevertheless, as we will see later, the resulting assumption is still useful for assessing the impact of false positives on clustering in networks.

Based on this assumption we have that

P(∨∈E2|∨∉E1)=∑u∈Vu≠v∑w∈Vw≠v,u[P((u,v)∈E2|(u,v)∉E1)⋅P((v,w)∈E2|(v,w)∉E1)]≈∑u,w∈V[P((u,v)∈E2|(u,v)∉E1)⋅P((v,w)∈E2|(v,w)∉E1)].     (16)
 MathType@MTEF@5@5@+=feaafiart1ev1aaatCvAUfKttLearuWrP9MDH5MBPbIqV92AaeXatLxBI9gBaebbnrfifHhDYfgasaacH8akY=wiFfYdH8Gipec8Eeeu0xXdbba9frFj0=OqFfea0dXdd9vqai=hGuQ8kuc9pgc9s8qqaq=dirpe0xb9q8qiLsFr0=vr0=vr0dc8meaabaqaciaacaGaaeqabaqabeGadaaakeaafaqabeGadaaabaGaemiuaaLaeiikaGIaeyikIOTaeyicI4Saemyrau0aaWbaaSqabeaacqaIYaGmaaGccqGG8baFcqGHOiI2cqGHjiYZcqWGfbqrdaahaaWcbeqaaiabigdaXaaakiabcMcaPaqaaiabg2da9aqaamaaqafabaWaaabuaeaacqGGBbWwcqWGqbaucqGGOaakcqGGOaakcqWG1bqDcqGGSaalcqWG2bGDcqGGPaqkcqGHiiIZcqWGfbqrdaahaaWcbeqaaiabikdaYaaakiabcYha8jabcIcaOiabdwha1jabcYcaSiabdAha2jabcMcaPiabgMGiplabdweafnaaCaaaleqabaGaeGymaedaaOGaeiykaKIaeyyXICTaemiuaaLaeiikaGIaeiikaGIaemODayNaeiilaWIaem4DaCNaeiykaKIaeyicI4Saemyrau0aaWbaaSqabeaacqaIYaGmaaGccqGG8baFcqGGOaakcqWG2bGDcqGGSaalcqWG3bWDcqGGPaqkcqGHjiYZcqWGfbqrdaahaaWcbeqaaiabigdaXaaakiabcMcaPiabc2faDbWceaqabeaacqWG3bWDcqGHiiIZcqWGwbGvaeaacqWG3bWDcqGHGjsUcqWG2bGDcqGGSaalcqWG1bqDaaqab0GaeyyeIuoaaSabaeqabaGaemyDauNaeyicI4SaemOvayfabaGaemyDauNaeyiyIKRaemODayhaaeqaniabggHiLdaakeaaaeaacqGHijYUaeaadaaeqbqaaiabcUfaBjabdcfaqjabcIcaOiabcIcaOiabdwha1jabcYcaSiabdAha2jabcMcaPiabgIGiolabdweafnaaCaaaleqabaGaeGOmaidaaOGaeiiFaWNaeiikaGIaemyDauNaeiilaWIaemODayNaeiykaKIaeyycI8Saemyrau0aaWbaaSqabeaacqaIXaqmaaGccqGGPaqkcqGHflY1cqWGqbaucqGGOaakcqGGOaakcqWG2bGDcqGGSaalcqWG3bWDcqGGPaqkcqGHiiIZcqWGfbqrdaahaaWcbeqaaiabikdaYaaakiabcYha8jabcIcaOiabdAha2jabcYcaSiabdEha3jabcMcaPiabgMGiplabdweafnaaCaaaleqabaGaeGymaedaaOGaeiykaKIaeiyxa0LaeiOla4caleaacqWG1bqDcqGGSaalcqWG3bWDcqGHiiIZcqWGwbGvaeqaniabggHiLdaaaOGaaCzcaiaaxMaadaqadaqaaiabigdaXiabiAda2aGaayjkaiaawMcaaaaa@CA4B@

*P*(∇ ∈ *E*^2^|∇ ∉ *E*^1^) can be rewritten similarly. Since all nodes have been selected as baits we have for each pair *u *and *v *that *P*((*u, v*) ∈ *E*^2^|(*u, v*) ∉ *E*^1^) = *ω*(*u, v*)(2 - *ω*(*u, v*)) ≈ 2*ω*(*u, v*). Hence, equations (15), (16) and (4) result in

Cv2≈∑u,w∈V2ω(u,v)2ω(v,w)2ω(u,w)∑u,w∈V2ω(u,v)2ω(v,w)=2θ∑u∈V(ku+ι)∑u,w∈V(ku+ι)2(kw+ι)2∑u,w∈V(ku+ι)(kw+ι)=2θ∑u∈V(ku+ι)∑u∈V(ku+ι)2∑w∈V(kw+ι)2∑u∈V(ku+ι)∑w∈V(kw+ι)=2θ(∑u∈V(ku+ι)2)2(∑u∈V(ku+ι))3:=2θξ     (17)
 MathType@MTEF@5@5@+=feaafiart1ev1aaatCvAUfKttLearuWrP9MDH5MBPbIqV92AaeXatLxBI9gBaebbnrfifHhDYfgasaacH8akY=wiFfYdH8Gipec8Eeeu0xXdbba9frFj0=OqFfea0dXdd9vqai=hGuQ8kuc9pgc9s8qqaq=dirpe0xb9q8qiLsFr0=vr0=vr0dc8meaabaqaciaacaGaaeqabaqabeGadaaakeaafaqadeabbaaaaeaacqWGdbWqdaqhaaWcbaGaemODayhabaGaeGOmaidaaOGaeyisIS7aaSaaaeaadaaeqaqaaiabikdaYGGaciab=L8a3jabcIcaOiabdwha1jabcYcaSiabdAha2bWcbaGaemyDauNaeiilaWIaem4DaCNaeyicI4SaemOvayfabeqdcqGHris5aOGaeiykaKIaeGOmaiJae8xYdCNaeiikaGIaemODayNaeiilaWIaem4DaCNaeiykaKIaeGOmaiJae8xYdCNaeiikaGIaemyDauNaeiilaWIaem4DaCNaeiykaKcabaWaaabeaeaacqaIYaGmcqWFjpWDcqGGOaakcqWG1bqDcqGGSaalcqWG2bGDcqGGPaqkcqaIYaGmcqWFjpWDcqGGOaakcqWG2bGDcqGGSaalcqWG3bWDcqGGPaqkaSqaaiabdwha1jabcYcaSiabdEha3jabgIGiolabdAfawbqab0GaeyyeIuoaaaaakeaacqGH9aqpdaWcaaqaaiabikdaYiabeI7aXbqaamaaqababaGaeiikaGIaem4AaS2aaSbaaSqaaiabdwha1bqabaGccqGHRaWkcqWF5oqAcqGGPaqkaSqaaiabdwha1jabgIGiolabdAfawbqab0GaeyyeIuoaaaGcdaWcaaqaamaaqababaGaeiikaGIaem4AaS2aaSbaaSqaaiabdwha1bqabaGccqGHRaWkcqWF5oqAcqGGPaqkdaahaaWcbeqaaiabikdaYaaakiabcIcaOiabdUgaRnaaBaaaleaacqWG3bWDaeqaaOGaey4kaSIae8xUdKMaeiykaKYaaWbaaSqabeaacqaIYaGmaaaabaGaemyDauNaeiilaWIaem4DaCNaeyicI4SaemOvayfabeqdcqGHris5aaGcbaWaaabeaeaacqGGOaakcqWGRbWAdaWgaaWcbaGaemyDauhabeaakiabgUcaRiab=L7aPjabcMcaPiabcIcaOiabdUgaRnaaBaaaleaacqWG3bWDaeqaaOGaey4kaSIae8xUdKMaeiykaKcaleaacqWG1bqDcqGGSaalcqWG3bWDcqGHiiIZcqWGwbGvaeqaniabggHiLdaaaaGcbaGaeyypa0ZaaSaaaeaacqaIYaGmcqWF4oqCaeaadaaeqaqaaiabcIcaOiabdUgaRnaaBaaaleaacqWG1bqDaeqaaOGaey4kaSIae8xUdKMaeiykaKcaleaacqWG1bqDcqGHiiIZcqWGwbGvaeqaniabggHiLdaaaOWaaSaaaeaadaaeqaqaaiabcIcaOiabdUgaRnaaBaaaleaacqWG1bqDaeqaaOGaey4kaSIae8xUdKMaeiykaKYaaWbaaSqabeaacqaIYaGmaaGcdaaeqaqaaiabcIcaOiabdUgaRnaaBaaaleaacqWG3bWDaeqaaOGaey4kaSIae8xUdKMaeiykaKYaaWbaaSqabeaacqaIYaGmaaaabaGaem4DaCNaeyicI4SaemOvayfabeqdcqGHris5aaWcbaGaemyDauNaeyicI4SaemOvayfabeqdcqGHris5aaGcbaWaaabeaeaacqGGOaakcqWGRbWAdaWgaaWcbaGaemyDauhabeaakiabgUcaRiab=L7aPjabcMcaPmaaqababaGaeiikaGIaem4AaS2aaSbaaSqaaiabdEha3bqabaGccqGHRaWkcqWF5oqAcqGGPaqkaSqaaiabdEha3jabgIGiolabdAfawbqab0GaeyyeIuoaaSqaaiabdwha1jabgIGiolabdAfawbqab0GaeyyeIuoaaaaakeaacqGH9aqpcqaIYaGmcqWF4oqCdaWcaaqaamaabmaabaWaaabeaeaacqGGOaakcqWGRbWAdaWgaaWcbaGaemyDauhabeaakiabgUcaRiab=L7aPbWcbaGaemyDauNaeyicI4SaemOvayfabeqdcqGHris5aOGaeiykaKYaaWbaaSqabeaacqaIYaGmaaaakiaawIcacaGLPaaadaahaaWcbeqaaiabikdaYaaaaOqaamaabmaabaWaaabeaeaacqGGOaakcqWGRbWAdaWgaaWcbaGaemyDauhabeaakiabgUcaRiab=L7aPjabcMcaPaWcbaGaemyDauNaeyicI4SaemOvayfabeqdcqGHris5aaGccaGLOaGaayzkaaWaaWbaaSqabeaacqaIZaWmaaaaaOGaeiOoaOJaeyypa0JaeGOmaiJae8hUdeNae8NVdGhaaiaaxMaacaWLjaWaaeWaaeaacqaIXaqmcqaI3aWnaiaawIcacaGLPaaaaaa@2690@

As a consequence, we have that *C*^2 ^≈ 2*θξ*.

**Figure 10:**
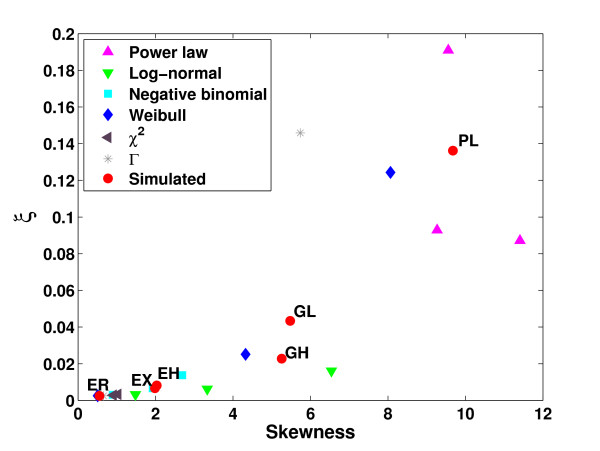
**Correlation between *ξ *and skewness**. Values of *ξ *were computed for a several topology models additional to the ones for which full simulations of our model were performed and plotted against the skewness of the corresponding networks. Topology models considered include power-law, log-normal, negative binomial, Weibull, *χ*^2 ^and Γ distributions with varying parameters. Parameters were tuned such that average degree values of 10 were obtained and results were averaged over 50 networks generated for each topology. The topology models for which complete simulations were performed are indicated in red.

Note that Σ_*u*∈*V*_(*k*_*u *_+ *ι*) = 2|*E*| + *ι*|*V*| is independent of the degree distribution whereas Σ_*u*∈*V*_(*k*_*u *_+ *ι*)^2 ^depends strongly on it. It is minimal if all nodes have the same average degree and maximal if all edges connect only one node to itself and the remaining nodes are singular, i.e. without connections. Accordingly, for networks with approximately the same number of nodes and edges, *ξ *is highly correlated with the skewness of the degree distribution (see also Figure 10).

The skewness of a network also allows us to assess how strongly the independence assumption is violated. As mentioned before, this assumption is only valid if the probability that an edge is added does not depend on the nodes it connects. In our model edges connecting two low-degree nodes to a hub are very likely whereas the probability that these nodes are then also connected is rather small. Accordingly, this has a negative effect on clustering and the more skewed a network, the more does the observed clustering coefficient deviate from the approximation. Furthermore, the observed clustering coefficients are on average smaller than the approximation. This is reasonable since *ξ *can become arbitrary large but the clustering coefficient is bounded from above by 1. As a consequence the minimum of 1 and 2*θξ *restricts the clustering coefficients observed on average in the simple case with *β *= 1 and *ε *= 0.

Of course, this simple scenario is insofar unrealistic as no experimental network should contain only wrong interactions and if it did it would be useless. However, as we will see later, the effect of false positive interactions on the clustering coefficient depends strongly on *ξ *also for *β *< 1 and *ε *> 0. In addition, the degree of randomness in clustering is also an important factor.

### Simulation results

To illustrate the effect of our model, corresponding simulations were performed for six different types of starting networks: (Poisson) random graphs (ER) [31], exponential networks with random (EX) and high (EH) clustering coefficients, power-law networks with random clustering coefficients (PL) and networks generated by a growth model [36] which aims at representing the evolution of protein interaction networks. In the last case, networks were generated with low (GL) and high (GH) clustering coefficients (see Additional File 1: Supplementary Figure 1 and Additional File 2: Supplementary Methods). To simulate the effect of the yeast two-hybrid methodology on the yeast interaction network, we generated networks for the described topologies, each containing 6,000 nodes (the approximate number of protein-encoding genes in yeast [37]) and average degree values of 5, 10 and 20. For each combination of network topology and average degree, 50 networks were generated and simulation results were averaged over these 50 networks.

#### Analysis of simulated clustering coefficients

**Figure 4:**
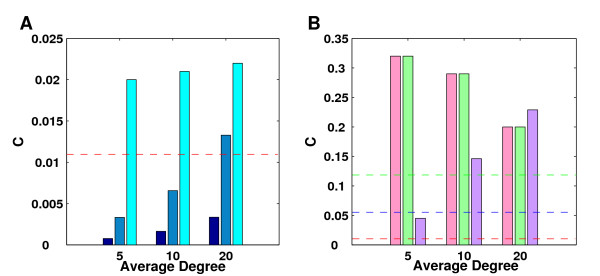
**Clustering coefficients of simulated networks**. For all six network topologies and average degree values, 50 networks were generated and clustering coefficients averaged over those 50 networks. In this figure the clustering coefficients of the ER, EX and GL (**A**, from left to right) and the EH, GH and PL (**B**) networks are compared against the minimum (red dashed line), average (blue) and maximum (green) clustering coefficient observed in experimental Y2H networks. For the EH and GH networks parameters were set such that both networks have approximately the same clustering coefficients.

The observed clustering coefficients for the generated networks (Figure 4) vary greatly between network topologies and average degree values. With the exception of the EH and GH networks which have been created specifically to show high clustering, two dependencies can be observed. The clustering coefficients are highly correlated with the average degree of the networks but also with the asymmetry of the degree distribution. Since random ER graphs follow a Poisson distribution and thus are little skewed, they exhibit the lowest degree of clustering of any of the topologies. Compared to that, exponential networks have an increased tendency for high and low degree nodes. As a consequence, they tend to be higher clustered than the ER networks. Despite the fact, that the GL networks have lower clustering coefficients than expected randomly for the degree distribution, they still show higher clustering coefficients than the exponential networks due to their high degree of skewness. Even if the clustering coefficients of the GL networks are randomized by edge rewiring, they are still lower than the clustering coefficients of the highly skewed power-law networks (PL).

When comparing the clustering coefficients of the large-scale Y2H networks against the simulated topologies, we observe that all of the PPI networks show higher clustering than the random ER graphs. In general, the experimental networks have higher clustering coefficients than the exponential networks and even the GL networks. Only the human interaction network by Stelzl et al. is lower clustered than all GL networks and exponential networks with high average degree values. Accordingly, only the EH and GH networks and the PL networks with high average degrees exhibit clustering coefficients which exceed those of all experimental Y2H protein-protein interaction networks.

These results as such do not exclude any of the topologies. However, when considering the effect of the different types of measurement errors on clustering coefficients, one should always keep in mind the original clustering coefficients we are starting from. In the following, the different effects of false negative and false positive interactions are again considered separately from each other.

#### Missing interactions

**Figure 5:**
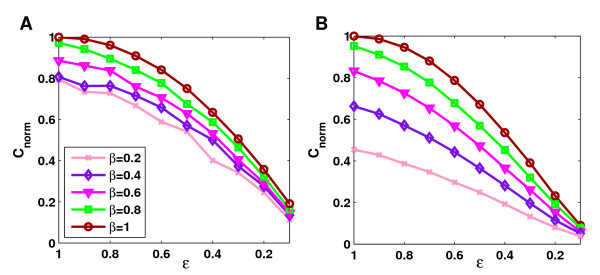
**Effect of sampling for limited coverage rates**. This figure demonstrates the impact of limited sampling on the average clustering coefficient for coverage rates below one. To illustrate differences between randomly and highly clustered networks, normalized clustering coefficients are depicted for the PL (**A**) and the EH (**B**) networks with average degree of 10. The highly clustered EH networks are affected to a much greater degree by low bait coverage rates than the randomly clustered PL networks. Clustering coefficients were normalized by dividing by the original clustering coefficients.

Our theoretical results predict that both limited bait coverage and limited edge coverage lower clustering coefficients significantly regardless of network topology and average degree. We have shown previously [28] in simulations that this prediction indeed holds for the ER, EX, GL and GH networks. Our extended simulations show that the EH and PL networks are affected similarly by false negative interactions (see Figure 5 and Additional file 1: Supplementary Figure 2). Here, to illustrate the dramatic decrease in clustering due to false negatives, clustering coefficients of the sampled networks were normalized by dividing by the original clustering coefficients.

We thus can confirm the observation from [28] that for all topology models the clustering coefficients of the sampled network are significantly lower than the clustering coefficients of the original networks for any value of bait or edge coverage. Furthermore, since we now considered more than one highly clustered network, we can draw conclusions about similarities between the randomly or less than randomly clustered ER, EX, PL and GL networks on the one hand and the more than randomly clustered EH and GH networks on the other hand. For all network topologies the effect of limited bait coverage is less severe than the effect of limited edge coverage. Yet, whereas limited bait coverage affects clustering in the ER, EX, PL and GL networks only to a minor degree, the effect on the highly clustered EH and PH networks is substantial. The differences between the two groups can be seen in Figure 5 for the PL and EH networks. Even at *ε *= 1, clustering coefficients in the EH networks are significantly smaller for small values of *β *than in the PL networks. For instance, at *β *= 0.2 they are only about half as high.

This observation is surprising since in our analytical derivations no such difference was observed. Nevertheless, it can be easily explained. In our derivations clustering coefficients were treated as continuous variables, whereas effectively they behave in a discrete manner since an edge can either exist or not. If we only consider nodes for which clustering coefficients before and after the simulation are greater than 0, no differences between highly and randomly clustered networks are observed. The differences observed are due to nodes for which *C*_*v *_> 0 and Cv1
 MathType@MTEF@5@5@+=feaafiart1ev1aaatCvAUfKttLearuWrP9MDH5MBPbIqV92AaeXatLxBI9gBaebbnrfifHhDYfgasaacH8akY=wiFfYdH8Gipec8Eeeu0xXdbba9frFj0=OqFfea0dXdd9vqai=hGuQ8kuc9pgc9s8qqaq=dirpe0xb9q8qiLsFr0=vr0=vr0dc8meaabaqaciaacaGaaeqabaqabeGadaaakeaacqWGdbWqdaqhaaWcbaGaemODayhabaGaeGymaedaaaaa@304D@ = 0 and nodes for which *C*_*v *_= 0 and Cv1
 MathType@MTEF@5@5@+=feaafiart1ev1aaatCvAUfKttLearuWrP9MDH5MBPbIqV92AaeXatLxBI9gBaebbnrfifHhDYfgasaacH8akY=wiFfYdH8Gipec8Eeeu0xXdbba9frFj0=OqFfea0dXdd9vqai=hGuQ8kuc9pgc9s8qqaq=dirpe0xb9q8qiLsFr0=vr0=vr0dc8meaabaqaciaacaGaaeqabaqabeGadaaakeaacqWGdbWqdaqhaaWcbaGaemODayhabaGaeGymaedaaaaa@304D@ = 0. In the first case, clustering coefficients decrease dramatically and stronger than expected, in the second case they do not decrease at all. In highly clustered networks the first type of nodes is much more common than in randomly clustered networks, whereas the second type of nodes is rarer. Accordingly, while for randomly clustered networks the effects on the two types of nodes cancel each other to a large degree, there is an excess of the first type of nodes in highly clustered networks. This leads to the stronger reduction in clustering coefficients observed.

#### Spurious interactions

**Figure 6:**
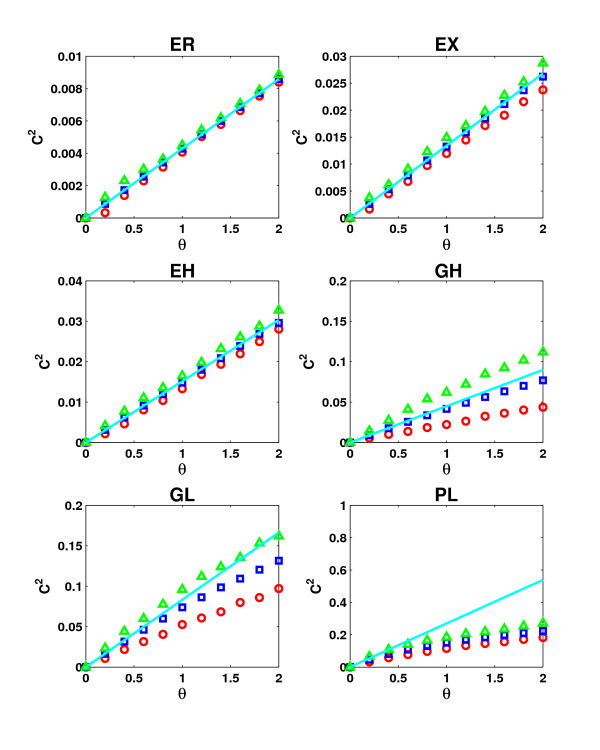
**Comparison between approximation and simulation of false positive interactions**. The minium (red), average (blue) and maximum (green) clustering coefficients obtained in 50 simulations of false positive interactions with *β *= 1 and *ε *= 0 are compared against the clustering coefficients predicted by the approximation 2*θξ *for networks of average degree 10. Topology models are sorted according to increasing skewness from top left to bottom right to illustrate the increasing deviation from the approximation with network skewness.

We have seen previously, that for *β *= 1 and *ε *= 0 the average clustering coefficient is expected to increase linearly with *θ *which is also confirmed in part by our simulations (Figure 6). However, for high values of *θ *a deviation from the linear behavior can be observed which leads to a slower increase. As mentioned before, this is due to the violation of the independence assumption. This violation leads to an ever stronger deviation with increasing skewness in the network. In Figure 6, topology models are sorted according to skewness. Accordingly, we observe that the more skewed a topology is, the smaller are the values of *θ *at which the observed clustering coefficients start to deviate from the linear behavior. This effect is most pronounced for the power-law networks, for which *ξ *predicts the highest increase in clustering due to false positive interactions. The effective increase turns out to be significantly less than predicted but is still much higher than for the other topologies, in particular for small values of *θ*.

**Figure 7:**
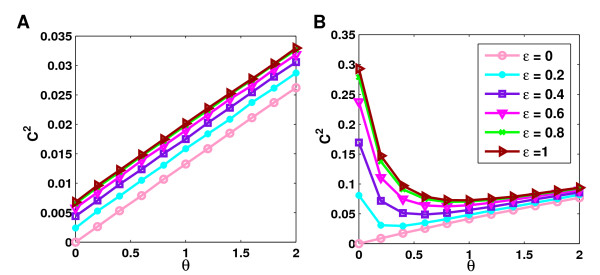
**Influence of spurious interactions on clustering**. Spurious interactions can influence average clustering coefficients in two ways depending on the degree of clustering in the network. In randomly (e.g. EX in **A**) or less than randomly clustered networks clustering coefficients can be increased by attaching false positive interactions. In highly clustered networks (e.g. GH in **B**), clustering coefficients are – at least for reasonable error rates – decreased. Here, average degree values were fixed at 10 and bait coverage at 1.

So far, edge coverage was restricted to 0. Figure 7 (see also Additional file 1: Supplementary Figure 3) illustrates the effect of different values of *ε *(but constant *β *= 1) and increasing *θ *on the clustering coefficient. For *ε *> 0, the effect on the clustering coefficient depends strongly not on the topology but on the degree of randomness in clustering. For two of the randomly clustered networks (ER and EX), the clustering coefficients increase linearly with *θ *for any *ε*. Indeed, if *C *is the original clustering coefficient, the resulting clustering coefficient *C*^2 ^can be approximated by 2*ξθ *+ *ε*(2 - *ε*)*C*. As before, the PL networks deviate from this behavior and clustering coefficients for higher values of *ε *increase more slowly than predicted. Furthermore, the higher *ε*, the lower is the rate of increase. As a consequence, the curves for *ε *= 0 and *ε *= 1 move towards each other for increasing *θ*.

For networks clustered less than randomly (GL), the average clustering coefficients for higher values of *ε *increase stronger than linearly at the beginning, until random clustering is reached in the network. From this point on a similar behavior is observed as for the randomly clustered networks. The contrary effect is found for highly clustered networks (EH and GH). In this case the clustering coefficients are reduced significantly by preferential attachment of false positives. Only when random clustering is reached in the network, clustering coefficients increase again depending on the value of *ξ *and thus on the asymmetry in the network. Nevertheless, the decrease in clustering due to missing interactions in these highly clustered networks can only be compensated for by very high error rates.

**Figure 8:**
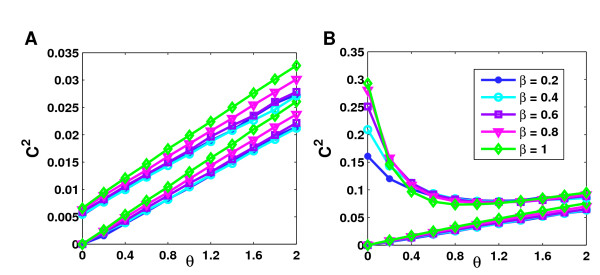
**Effect of spurious interactions at different values of bait coverage**. This figure illustrates the differences which are observed for values of bait coverage smaller than 1 and for edge coverage rates of 1 and 0. Again the EX (**A**) and GH (**B**) networks of average degree 10 were chosen. We observe that clustering coefficients for smaller values of *β *are, in general, slightly smaller then for *β *= 1, but the differences are minor.

For *β *< 1, the effect of erroneous interactions on the average clustering coefficient is similar to the case in which all proteins are selected as baits (see Figure 8). Clustering coefficients can be increased as well for randomly clustered networks, but the increase turns out to be slightly less than before. A possible explanation for this observation might be that wrong interactions are only ever added between baits and preys (or other baits) but never between preys. Thus, for small values of *β*, baits are often connected to two preys which by definition of the model can never be connected. This results in smaller clustering in the network.

**Figure 9:**
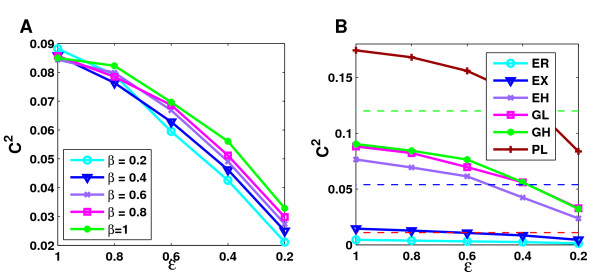
**Clustering coefficients at a fixed false positive rate**. The combined effect of the different error mechanisms were analyzed by setting the false positive rate at a fixed value of 50%. For each combination of *β *and *ε*, *θ *was then chosen accordingly. **A **shows the resulting clustering coefficients at different bait coverage rates for the GL networks and **B **for each topology the maximum over the averages obtained for the different values of *β *considered. Minimum, average and maximum clustering coefficients observed in the real Y2H experiments are indicated by red, blue and green dashed lines.

To illustrate the combined effect of different parameter values for the model, simulations were performed in which for each value of *β *and *ε*, *θ *was chosen such that the same fixed false positive rate of 50% was obtained (see Figure 9 and Additional file 1: Supplementary Figure 4). In these simulations several observations could be made. First, of course, clustering coefficients tend to be highest for high values of edge coverage and decrease with edge coverage. Second, for the ER, EX and GL networks the clustering coefficients obtained are higher than the clustering coefficients in the original simulated networks even for small edge coverage rates, whereas for the PL networks this requires higher edge coverage. On the contrary, in the EH and GH networks the resulting clustering coefficients are always significantly smaller than the original clustering coefficients for the given false positive rate. Here, only extremely high values of *θ *and thus the false positive rate could increase clustering coefficients beyond the original value. In both cases, this is due to the different effects of false positive interactions on randomly and highly clustered networks. Furthermore, clustering coefficients tend to be similar for different values of *β*. The ER and EX networks show only minor differences, whereas stronger differences can be observed for the other network types. In this case, the differences are most pronounced for the highly clustered EH networks.

In order to compare the effects on clustering at 50% false positive rate between topologies models, we computed for each topology the maximum over the averages for different values of *β *(see Figure 9B). Clustering coefficients from real Y2H experiments are also indicated. As can be seen, even by introducing false positive interactions clustering coefficients in ER and EX networks cannot be increased sufficiently to explain at least most of the observed Y2H networks by such a topology. The only topologies for which realistic clustering coefficients are observed are thus highly clustered exponential networks, the growth models and the power-law networks. Note that although EH networks were created with approximately the same clustering coefficients as the GH networks, the final clustering coefficients observed for these networks are nevertheless smaller than for the GH network. This can be explained by the fact that the increase in clustering for high *θ *as well as the lowest level up to which clustering coefficients decrease for smaller *θ *depend strongly on the skewness of the network topology which is higher for the GH networks than the EH networks.

Although we considered several possible topology models, there is an infinite number of possible topologies for which we did not perform simulations of our model. Nevertheless, the results presented above can be transfered to other topologies by taking into account the skewness of these models. If networks are clustered randomly, the clustering coefficients observed depend on the skewness of the corresponding degree distribution. Thus, highly skewed networks have high random clustering coefficients whereas slightly skewed or symmetric distributions exhibit very small clustering coefficients which are, in particular, smaller than clustering coefficients observed in real Y2H interaction networks. We have shown that missing interactions decrease these clustering coefficients even further. Only false positive interactions can increase clustering again in randomly clustered networks depending on the network topology. In networks clustered higher than randomly, clustering coefficients are decreased even by false positive interactions. Our simulation suggest that *ξ*, although not a perfect approximation at least restricts from above the clustering coefficients observed in randomly clustered networks under the influence of false positive interactions. The higher *ξ*, the higher the increase in clustering due to false positive interactions in randomly clustered networks, although returns diminish with increasing *ξ*. Accordingly, we computed the values of *ξ *for a range of additional topologies and plotted them against the skewness of the corresponding networks (see Figure 10). For each topology model we generated 50 networks and averaged over the corresponding *ξ *and skewness values. As can be seen, *ξ *is highly correlated to the skewness of network models. Accordingly, false positive interactions can only increase the clustering coefficients of networks sufficiently which follow a highly skewed degree distribution.

## Discussion

In a recent report, Han et al. [27] raised the possibility that the apparent scale-free topology of experimental Y2H interaction networks is due to distorting effects of limited sampling in large-scale experiments and that by examining the degree distribution alone, the topology of the experimental interaction networks cannot be safely extrapolated to the complete interactome. In this context, our results indicate that based on additional topological characteristics such as the clustering coefficient, the range of possible topologies can be narrowed. Thus, although current large-scale PPI networks represent only a fraction of the interactomes, they can nevertheless be used to draw some inferences to the topological characteristics of the complete interactomes.

We have shown both analytically and in simulations that sampling with limited bait and edge coverage lowers the clustering coefficient tremendously for any of the examined network topologies. This result has several implications concerning the topology of the complete interactomes. In this setting, the clustering coefficients observed in protein-protein interaction maps derived with high-throughput methods provide a lower bound on the clustering coefficients observed in complete interactomes. This furthermore suggests that the interactomes are highly clustered, much more than the simple random graph (ER), exponential (EX) or growth networks (GL). Accordingly, such topologies can be ruled out if the effect of spurious interactions is ignored. These findings do not eliminate the possibility that the original networks show a highly clustered topology different from a power-law topology.

Notwithstanding these considerations, we can use the relationship between clustering coefficients and bait and edge coverage to estimate the amount of error involved if we know both the original and resulting clustering coefficient and vice versa assess the original clustering coefficient based on the error rate and the observed clustering coefficient. In our simulations we found that in order to increase skewness in a network by limited sampling and thus to change the original distribution towards a power-law topology, bait coverage rates have to be lowered considerably. The degree to which they have to be lowered depends on the difference of the original topology to a power-law topology. Lowering edge coverage rates, on the other hand, does not have a sufficiently distorting effect. However, we have seen above that limited bait coverage leads to a significant reduction in clustering coefficients in highly clustered networks such as EH and GH. Thus, high original clustering coefficients would have to be assumed for the interactome, if the observed interaction networks were sampled from a highly clustered distribution which is significantly different from a power-law distribution (e.g. an exponential distribution). If such a high degree of clustering a appears unreasonable, the obvious conclusion is that the original interactome does in fact exhibit a power-law or a similar highly skewed topology.

We extended the sampling procedure described by Han et al. to cover the influence of false positive interactions on the topology of sampled networks. This leads to a more realistic model of Y2H experiments since spurious interactions are observed regularly in large-scale experiments. Without considering false positives the effect of sampling on the topology and the clustering coefficient might be underestimated or misinterpreted. In our model, interactions are introduced by a preferential attachment scenario in which the probability of obtaining wrong interactions depends on the degree of the nodes participating in an interaction. Furthermore, baits are more likely to acquire interactions than preys. This introduces a possible source of degree asymmetry in the model which is a consequence of the experimental set-up and not the topology of the network.

Based on the extended model, the conclusions drawn from the simple sampling model can be generalized. Preferential attachment of false positive interactions increases the clustering coefficients of networks which are clustered randomly (ER, EX and PL) or less than randomly (GL), but decreases the clustering coefficient for networks which are clustered higher than randomly (EH and GH) except for extremely high error rates. As a consequence, random graph and randomly clustered exponential networks still can be excluded confidently since unreasonably high error rates would have to be assumed to explain the clustering coefficients observed. Contrary to that, clustering coefficients of GL and even more so of PL networks can be increased sufficiently by introducing wrong interactions to explain at least most of the observed clustering coefficients. Indeed at 50% false positive rate, similar clustering coefficients can be obtained for the GL networks as for the highly clustered exponential (EH) and growth networks (GH) whose clustering coefficients are decreased by wrong interactions.

Accordingly, our simulation results suggest that the interactome either follows a power-law or similarly skewed degree distribution or is highly clustered. Nevertheless, we can make the same argument as before, that changing e.g. an exponential towards a power-law topology requires small bait coverage rates and consequently high clustering coefficients in the original network.

For random and semi-preferential attachment, estimates for the expected increase in clustering for *β *= 1 and *ε *= 0 can be derived in the same way as for preferential attachment. However, the rate of increase is smaller for both random and semi-preferential attachment than for preferential attachment. Accordingly, at *ε *> 0, clustering coefficients can decrease even for randomly clustered networks. In the semi-preferential model, this is only the case for highly skewed networks such as the PL networks. In the random attachment scenario, this happens even for the slightly skewed exponential networks.

Simulations of false negative and positive interactions were only performed for networks with average degree values of 5, 10 and 20. Higher average degree values in the original networks lead to higher random clustering coefficients in the original networks and thus in the sampled networks. Hence, one might argue that the above conclusions are invalid if original average degrees only have to be increased sufficiently. Effectively, such considerations are limited by what is actually observed in experimental networks. This can be illustrated by the following example. Suppose, a matrix Y2H screen (*β *= 1) results in a network with average degree k¯
 MathType@MTEF@5@5@+=feaafiart1ev1aaatCvAUfKttLearuWrP9MDH5MBPbIqV92AaeXatLxBI9gBaebbnrfifHhDYfgasaacH8akY=wiFfYdH8Gipec8Eeeu0xXdbba9frFj0=OqFfea0dXdd9vqai=hGuQ8kuc9pgc9s8qqaq=dirpe0xb9q8qiLsFr0=vr0=vr0dc8meaabaqaciaacaGaaeqabaqabeGadaaakeaacuWGRbWAgaqeaaaa@2E23@' of 5 and the false positive rate is estimated to be 50%. Then, edge coverage and original average degree k¯
 MathType@MTEF@5@5@+=feaafiart1ev1aaatCvAUfKttLearuWrP9MDH5MBPbIqV92AaeXatLxBI9gBaebbnrfifHhDYfgasaacH8akY=wiFfYdH8Gipec8Eeeu0xXdbba9frFj0=OqFfea0dXdd9vqai=hGuQ8kuc9pgc9s8qqaq=dirpe0xb9q8qiLsFr0=vr0=vr0dc8meaabaqaciaacaGaaeqabaqabeGadaaakeaacuWGRbWAgaqeaaaa@2E23@ are related by the formula

ε(2−ε)=k¯′2k¯.     (18)
 MathType@MTEF@5@5@+=feaafiart1ev1aaatCvAUfKttLearuWrP9MDH5MBPbIqV92AaeXatLxBI9gBaebbnrfifHhDYfgasaacH8akY=wiFfYdH8Gipec8Eeeu0xXdbba9frFj0=OqFfea0dXdd9vqai=hGuQ8kuc9pgc9s8qqaq=dirpe0xb9q8qiLsFr0=vr0=vr0dc8meaabaqaciaacaGaaeqabaqabeGadaaakeaaiiGacqWF1oqzcqGGOaakcqaIYaGmcqGHsislcqWF1oqzcqGGPaqkcqGH9aqpdaWcaaqaaiqbdUgaRzaaryaafaaabaGaeGOmaiJafm4AaSMbaebaaaGaeiOla4IaaCzcaiaaxMaadaqadaqaaiabigdaXiabiIda4aGaayjkaiaawMcaaaaa@3E2D@

Thus, if k¯
 MathType@MTEF@5@5@+=feaafiart1ev1aaatCvAUfKttLearuWrP9MDH5MBPbIqV92AaeXatLxBI9gBaebbnrfifHhDYfgasaacH8akY=wiFfYdH8Gipec8Eeeu0xXdbba9frFj0=OqFfea0dXdd9vqai=hGuQ8kuc9pgc9s8qqaq=dirpe0xb9q8qiLsFr0=vr0=vr0dc8meaabaqaciaacaGaaeqabaqabeGadaaakeaacuWGRbWAgaqeaaaa@2E23@ = 2.5, *ε *is approximately 1. For k¯
 MathType@MTEF@5@5@+=feaafiart1ev1aaatCvAUfKttLearuWrP9MDH5MBPbIqV92AaeXatLxBI9gBaebbnrfifHhDYfgasaacH8akY=wiFfYdH8Gipec8Eeeu0xXdbba9frFj0=OqFfea0dXdd9vqai=hGuQ8kuc9pgc9s8qqaq=dirpe0xb9q8qiLsFr0=vr0=vr0dc8meaabaqaciaacaGaaeqabaqabeGadaaakeaacuWGRbWAgaqeaaaa@2E23@ = 5 it is 0.29, for k¯
 MathType@MTEF@5@5@+=feaafiart1ev1aaatCvAUfKttLearuWrP9MDH5MBPbIqV92AaeXatLxBI9gBaebbnrfifHhDYfgasaacH8akY=wiFfYdH8Gipec8Eeeu0xXdbba9frFj0=OqFfea0dXdd9vqai=hGuQ8kuc9pgc9s8qqaq=dirpe0xb9q8qiLsFr0=vr0=vr0dc8meaabaqaciaacaGaaeqabaqabeGadaaakeaacuWGRbWAgaqeaaaa@2E23@ = 10 it is 0.13, and so on. Accordingly, high average degree values can only be assumed if coverage rates are small. This on the other hand implies that although original clustering coefficients might be higher, the clustering coefficients resulting from the experiment are very small due to the low coverage rates.

The error mechanisms we proposed for our model are fairly simple and require few assumptions. Of course, many other error mechanisms are also possible (see e.g. [38]) and we can never be sure that the way interactions are added describes the processes occurring in large-scale experiments accurately. As a consequence, the preferential attachment scenario was chosen to simulate the worst case in which false positive interactions also promote a scale-free topology in experimental networks regardless of the original topology. We showed that, even when assuming this worst case, conclusions can still be drawn to the topology of the interactome. Nevertheless, our results do not only apply to our model but can be generalized to a wider range of error mechanisms. Randomly removing edges from a network in general reduces clustering coefficients in this network. On the other hand, adding edges to a network increases clustering only if the probability that triangles are created is at least as high as the probability that triangles exist in the original network. Random error processes, however, create most likely also random clustering coefficients. Accordingly, if the original networks are clustered higher than randomly, clustering coefficients are expected to decrease.

## Conclusion

We conclude that measurement errors in large-scale experiments affect several aspects of the network topology apart from the degree distribution. The impact of the experimental set-up on these other characteristics may be used to infer the topology of the complete interactome. In this article, we focused on the average clustering coefficient to evaluate the likelihood of different topological models for the interactome. Our analytical and simulation results indicate that some of the suggested topologies are highly unlikely and can be excluded with high confidence. Although only a selection of possible topology models was discussed in this article, we have shown how the results can be transfered to other topologies as well. With the help of additional topological characteristics and constraints, such as e.g. attack tolerance, our results might be extended to further resolve the topology of the interactome. Of course, the most effective and most conclusive way to achieve this aim, is to increase the coverage of the interactome by both many more experiments and by improving the false positive and false negative rates of large-scale methods. However, until this is realized, useful conclusions can still be drawn from modeling sampling effects.

## Authors' contributions

CF derived the theoretical results and implemented the simulation model. RZ participated in the design of the study and interpreting the results. The manuscript was written by CF and RZ. All authors read and approved the final manuscript.

## Supplementary Material

Additional File 1 **Supplementary Figures**. This file provides figures of the simulation results for all topology models and average degree values. Click here for file

Additional File 2 **Supplementary Methods**. This file contains a description of the methods used for generating networks with different topologies. Click here for file

## References

[B1] Uetz P, Giot L, Cagney G, Mansfield TA, Judson RS, Knight JR, Lockshon D, Narayan V, Srinivasan M, Pochart P, Qureshi-Emili A, Li Y, Godwin B, Conover D, Kalbfleisch T, Vijayadamodar G, Yang M, Johnston M, Fields S, Rothberg JM (2000). A comprehensive analysis of protein-protein interactions in Saccharomyces cerevisiae. Nature.

[B2] Ito T, Chiba T, Ozawa R, Yoshida M, Hattori M, Sakaki Y (2001). A comprehensive two-hybrid analysis to explore the yeast protein interactome. Proc Natl Acad Sci USA.

[B3] Li S, Armstrong CM, Bertin N, Ge H, Milstein S, Boxem M, Vidalain PO, Han JDJ, Chesneau A, Hao T, Goldberg DS, Li N, Martinez M, Rual JF, Lamesch P, Xu L, Tewari M, Wong SL, Zhang LV, Berriz GF, Jacotot L, Vaglio P, Reboul J, Hirozane-Kishikawa T, Li Q, Gabel HW, Elewa A, Baumgartner B, Rose DJ, Yu H, Bosak S, Sequerra R, Fraser A, Mango SE, Saxton WM, Strome S, Heuvel SVD, Piano F, Vandenhaute J, Sardet C, Gerstein M, Doucette-Stamm L, Gunsalus KC, Harper JW, Cusick ME, Roth FP, Hill DE, Vidal M (2004). A map of the interactome network of the metazoan C. elegans. Science.

[B4] Giot L, Bader JS, Brouwer C, Chaudhuri A, Kuang B, Li Y, Hao YL, Ooi CE, Godwin B, Vitols E, Vijayadamodar G, Pochart P, Machineni H, Welsh M, Kong Y, Zerhusen B, Malcolm R, Varrone Z, Collis A, Minto M, Burgess S, McDaniel L, Stimpson E, Spriggs F, Williams J, Neurath K, Ioime N, Agee M, Voss E, Furtak K, Renzulli R, Aanensen N, Carrolla S, Bickelhaupt E, Lazovatsky Y, DaSilva A, Zhong J, Stanyon CA, Finley RL, White KP, Braverman M, Jarvie T, Gold S, Leach M, Knight J, Shimkets RA, McKenna MP, Chant J, Rothberg JM (2003). A protein interaction map of Drosophila melanogaster. Science.

[B5] LaCount DJ, Vignali M, Chettier R, Phansalkar A, Bell R, Hesselberth JR, Schoenfeld LW, Ota I, Sahasrabudhe S, Kurschner C, Fields S, Hughes RE (2005). A protein interaction network of the malaria parasite Plasmodium falciparum. Nature.

[B6] Rual JF, Venkatesan K, Hao T, Hirozane-Kishikawa T, Dricot A, Li N, Berriz GF, Gibbons FD, Dreze M, Ayivi-Guedehoussou N, Klitgord N, Simon C, Boxem M, Milstein S, Rosenberg J, Goldberg DS, Zhang LV, Wong SL, Franklin G, Li S, Albala JS, Lim J, Fraughton C, Llamosas E, Cevik S, Bex C, Lamesch P, Sikorski RS, Vandenhaute J, Zoghbi HY, Smolyar A, Bosak S, Sequerra R, Doucette-Stamm L, Cusick ME, Hill DE, Roth FP, Vidal M (2005). Towards a proteome-scale map of the human protein-protein interaction network. Nature.

[B7] Stelzl U, Worm U, Lalowski M, Haenig C, Brembeck FH, Goehler H, Stroedicke M, Zenkner M, Schoenherr A, Koeppen S, Timm J, Mintzlaff S, Abraham C, Bock N, Kietzmann S, Goedde A, Toksöz E, Droege A, Krobitsch S, Korn B, Birchmeier W, Lehrach H, Wanker EE (2005). A human protein-protein interaction network: a resource for annotating the proteome. Cell.

[B8] Ho Y, Gruhler A, Heilbut A, Bader GD, Moore L, Adams SL, Millar A, Taylor P, Bennett K, Boutilier K, Yang L, Wolting C, Donaldson I, Schandorff S, Shewnarane J, Vo M, Taggart J, Goudreault M, Muskat B, Alfarano C, Dewar D, Lin Z, Michalickova K, Willems AR, Sassi H, Nielsen PA, Rasmussen KJ, Andersen JR, Johansen LE, Hansen LH, Jespersen H, Podtelejnikov A, Nielsen E, Crawford J, Poulsen V, Sørensen BD, Matthiesen J, Hendrickson RG, Gleeson F, Pawson T, Moran MF, Durocher D, Mann M, Hogue CWV, Figeys D, Tyers M (2002). Systematic identification of protein complexes in Saccharomyces cerevisiae by mass spectrometry. Nature.

[B9] Gavin AC, Bösche M, Krause R, Grandi P, Marzioch M, Bauer A, Schultz J, Rick JM, Michon AM, Cruciat CM, Remor M, Höfert C, Schelder M, Brajenovic M, Ruffner H, Merino A, Klein K, Hudak M, Dickson D, Rudi T, Gnau V, Bauch A, Bastuck S, Huhse B, Leutwein C, Heurtier MA, Copley RR, Edelmann A, Querfurth E, Rybin V, Drewes G, Raida M, Bouwmeester T, Bork P, Seraphin B, Kuster B, Neubauer G, Superti-Furga G (2002). Functional organization of the yeast proteome by systematic analysis of protein complexes. Nature.

[B10] Gavin AC, Aloy P, Grandi P, Krause R, Boesche M, Marzioch M, Rau C, Jensen LJ, Bastuck S, Dümpelfeld B, Edelmann A, Heurtier MA, Hoffman V, Hoefert C, Klein K, Hudak M, Michon AM, Schelder M, Schirle M, Remor M, Rudi T, Hooper S, Bauer A, Bouwmeester T, Casari G, Drewes G, Neubauer G, Rick JM, Kuster B, Bork P, Russell RB, Superti-Furga G (2006). Proteome survey reveals modularity of the yeast cell machinery. Nature.

[B11] Krogan NJ, Cagney G, Yu H, Zhong G, Guo X, Ignatchenko A, Li J, Pu S, Datta N, Tikuisis AP, Punna T, Peregrín-Alvarez JM, Shales M, Zhang X, Davey M, Robinson MD, Paccanaro A, Bray JE, Sheung A, Beattie B, Richards DP, Canadien V, Lalev A, Mena F, Wong P, Starostine A, Canete MM, Vlasblom J, Wu S, Orsi C, Collins SR, Chandran S, Haw R, Rilstone JJ, Gandi K, Thompson NJ, Musso G, Onge PS, Ghanny S, Lam MHY, Butland G, Altaf-Ul AM, Kanaya S, Shilatifard A, O'Shea E, Weissman JS, Ingles CJ, Hughes TR, Parkinson J, Gerstein M, Wodak SJ, Emili A, Greenblatt JF (2006). Global landscape of protein complexes in the yeast Saccharomyces cerevisiae. Nature.

[B12] Deane CM, Salwínski L, Xenarios I, Eisenberg D (2002). Protein interactions: two methods for assessment of the reliability of high throughput observations. Mol Cell Proteomics.

[B13] Bader GD, Hogue CWV (2002). Analyzing yeast protein-protein interaction data obtained from different sources. Nat Biotechnol.

[B14] Bader JS, Chaudhuri A, Rothberg JM, Chant J (2004). Gaining confidence in high-throughput protein interaction networks. Nat Biotechnol.

[B15] Jeong H, Mason SP, Barabási AL, Oltvai ZN (2001). Lethality and centrality in protein networks. Nature.

[B16] Wagner A (2001). The yeast protein interaction network evolves rapidly and contains few redundant duplicate genes. Mol Biol Evol.

[B17] Eisenberg E, Levanon EY (2003). Preferential attachment in the protein network evolution. Phys Rev Lett.

[B18] Wuchty S (2004). Evolution and topology in the yeast protein interaction network. Genome Res.

[B19] Yook SH, Oltvai ZN, Barabási AL (2004). Functional and topological characterization of protein interaction networks. Proteomics.

[B20] Coulomb S, Bauer M, Bernard D, Marsolier-Kergoat MC (2005). Gene essentiality and the topology of protein interaction networks. Proc Biol Sci.

[B21] Dorogovtsev S, Mendes J (2002). Evolution of networks. Adv Phys.

[B22] Albert R, Barabasi AL (2002). Statistical mechanics of complex networks. Reviews of Modern Physics.

[B23] Newman M (2003). The structure and function of complex networks. SIAM Review.

[B24] Tanaka R, Yi TM, Doyle J (2005). Some protein interaction data do not exhibit power law statistics. FEBS Lett.

[B25] Przulj N, Corneil DG, Jurisica I (2004). Modeling interactome: scale-free or geometric?. Bioinformatics.

[B26] Stumpf MPH, Wiuf C, May RM (2005). Subnets of scale-free networks are not scale-free: sampling properties of networks. Proc Natl Acad Sci USA.

[B27] Han JDJ, Dupuy D, Bertin N, Cusick ME, Vidal M (2005). Effect of sampling on topology predictions of protein-protein interaction networks. Nat Biotechnol.

[B28] Friedel CC, Zimmer R (2006). Toward the complete interactome. Nat Biotechnol.

[B29] Barabási A, Albert R (1999). Emergence of scaling in random networks. Science.

[B30] Watts DJ, Strogatz SH (1998). Collective dynamics of 'small-world' networks. Nature.

[B31] Erdős P, Rényi A (1959). On random graphs. Publicationes Mathematicae.

[B32] Maslov S, Sneppen K (2002). Specificity and stability in topology of protein networks. Science.

[B33] Xenarios I, Salwínski L, Duan XJ, Higney P, Kim SM, Eisenberg D (2002). DIP, the Database of Interacting Proteins: a research tool for studying cellular networks of protein interactions. Nucleic Acids Res.

[B34] Chung F, Lu L (2002). The average distances in random graphs with given expected degrees. Proc Natl Acad Sci USA.

[B35] Legrain P, Selig L (2000). Genome-wide protein interaction maps using two-hybrid systems. FEBS Lett.

[B36] Vázquez A, Flammini A, Maritan A, Vespignani A (2003). Modeling of protein interaction networks. ComPlexUs.

[B37] Goffeau A, Barrell BG, Bussey H, Davis RW, Dujon B, Feldmann H, Galibert F, Hoheisel JD, Jacq C, Johnston M, Louis EJ, Mewes HW, Murakami Y, Philippsen P, Tettelin H, Oliver SG (1996). Life with 6000 genes. Science.

[B38] Lin N, Zhao H (2005). Are scale-free networks robust to measurement errors?. BMC Bioinformatics.

