# Supplementary material for: Inferring topology from clustering coefficients in protein-protein interaction networks
Source: BMC Bioinformatics. 2006 Nov 30;7:519. doi: 10.1186/1471-2105-7-519 (PMC1716184; doi:10.1186/1471-2105-7-519)
Supplement: Additional File 1 — Supplementary Figures. This file provides figures of the simulation results for all topology models and average degree values. [file 1471-2105-7-519-S1.pdf]

Supplementary Figures

Supplementary Figure 1 - Degree distributions for network models

Six topology models were simulated: Erdős-Rényi random graphs (ER) [1], exponential networks with random (EX) and high clustering coefficients (EH), growth networks with low (GL) and high clustering coefficients (GH) and power-law networks (PL) with random clustering coefficients. Average degree values $\bar{k}$ of 5, 10 and 20 were chosen. The size of the networks was fixed at 6000 nodes, which approximately corresponds to the number of protein-encoding genes in *S. cerevisiae* [2]. This figure shows the average degree distribution $P(k)$ for each combination of topology model and average degree.

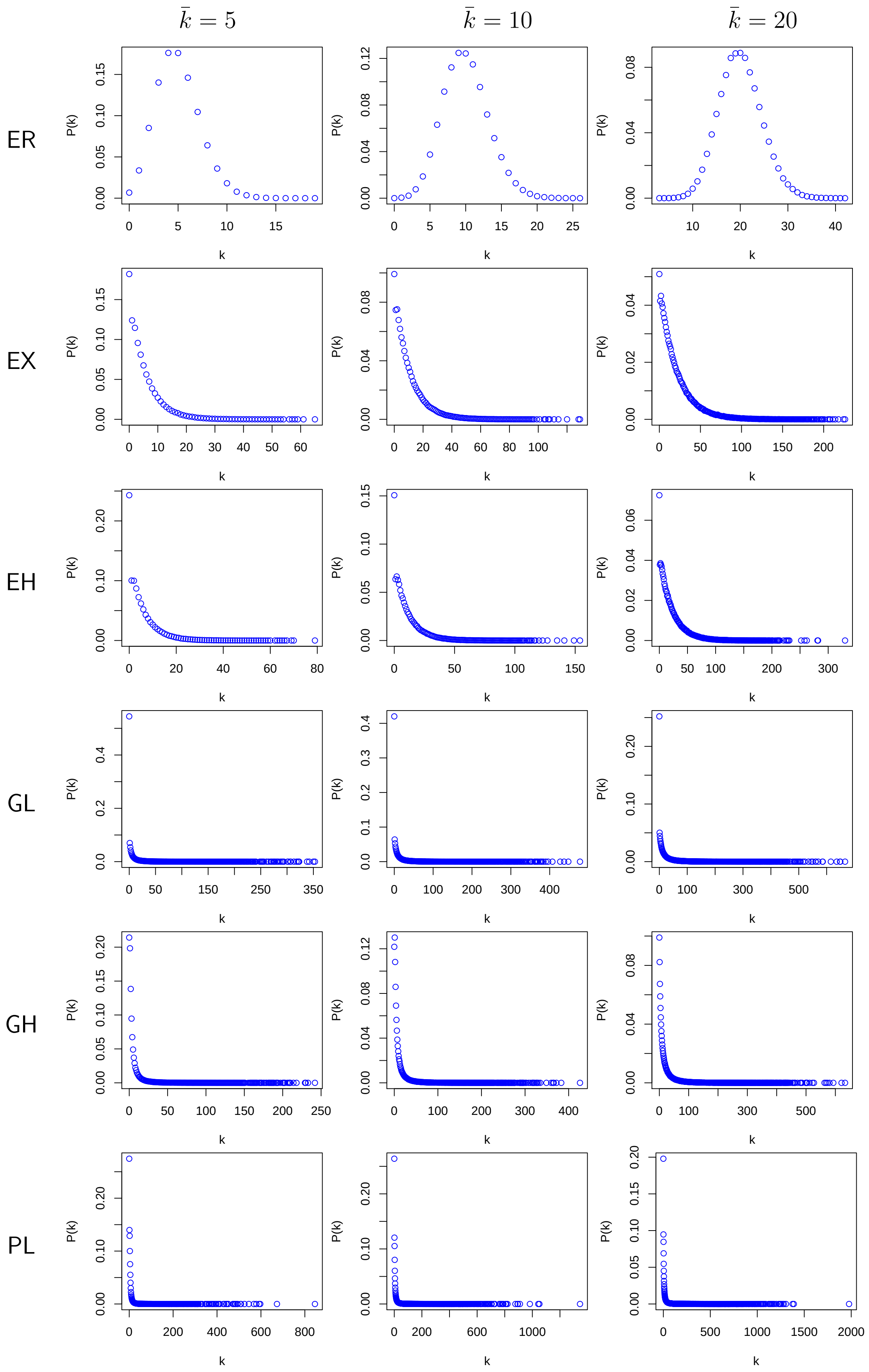

$\bar{k} = 5$
$\bar{k} = 10$
$\bar{k} = 20$
ER
EX
EH
GL
GH
PL
P(k)
k

Supplementary Figure 2 - Effect of limited sampling for all combinations of coverage rates

Simulations were performed for 50 networks containing 6000 nodes for each of the six topology models: Erdős-Rényi random graphs (ER), exponential networks with random (EX) and high (EH) clustering, growth with low (GL) and high clustering coefficients (GH) and power-law networks (PL). Networks were generated for average degree values of 5, 10 and 20 and sampled for discrete values of bait and edge coverage. This figure shows for the ER, EX, GL and GH networks the same data as in [3]. For comparison purposes, the results for these networks are nevertheless shown alongside the results for the EH and PL networks. Furthermore, a different layout was chosen to illustrate the differences between the highly and randomly clustered networks. Thus, the x coordinate represents values of ε and the y coordinate the normalized clustering coefficients (C_{norm}) which were normalized by dividing the average clustering coefficients of the sampled networks by the original average clustering coefficients. Network models are grouped according to the degree of randomness in clustering in the original networks to illustrate the differences between these groups. First we have the GL networks which are clustered less than randomly, then we have the randomly clustered ER, EX and PL networks and finally the highly clustered EH and GL networks.

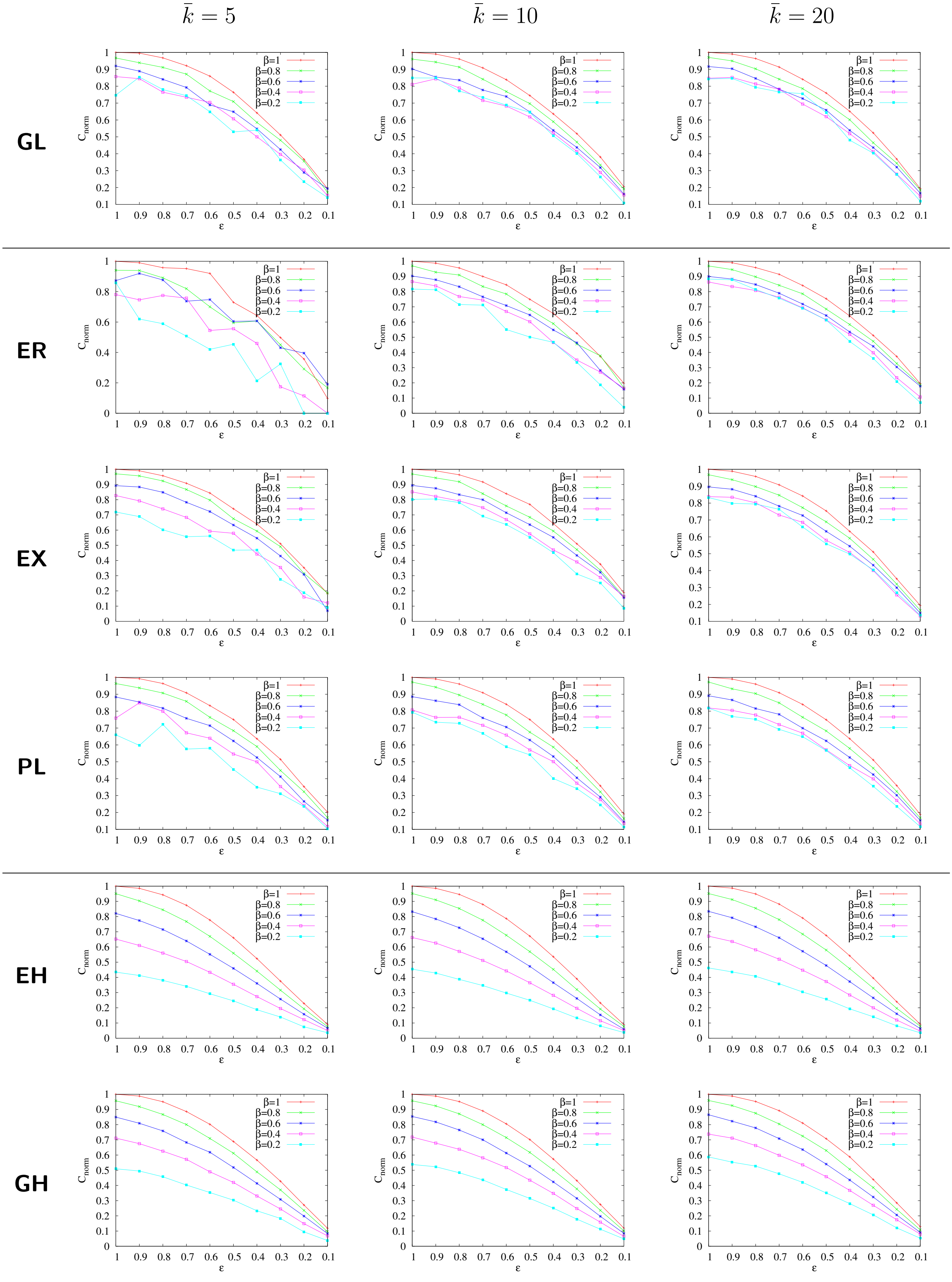

$\bar{k} = 5$
$\bar{k} = 10$
$\bar{k} = 20$
GL
ER
EX
PL
EH
GH
C_{norm}
ε
β=1
β=0.8
β=0.6
β=0.4
β=0.2

Supplementary Figure 3 - Influence of spurious interactions on clustering

This Figure illustrates how different topologies are affected by introducing wrong interactions into the networks. The x coordinate represents the error rate θ and the y coordinate the average clustering coefficients for different values of ε. For this purpose, β was set to one. Again, network models are grouped according to the degree of randomness in clustering in the original networks in order to illustrate the differences between these groups.

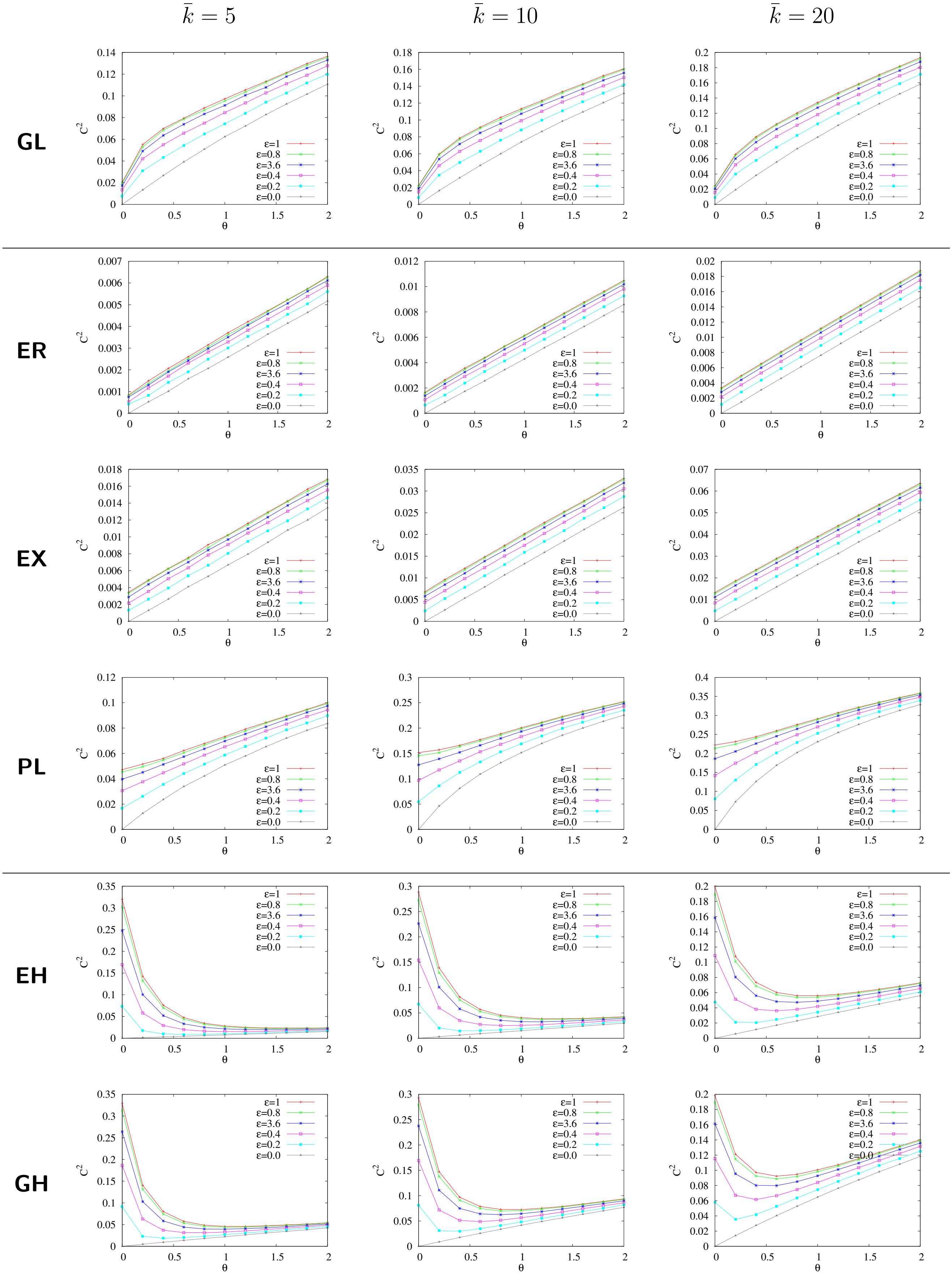
$\bar{k} = 5$
$\bar{k} = 10$
$\bar{k} = 20$
GL
ER
EX
PL
EH
GH
ε=1
ε=0.8
ε=3.6
ε=0.4
ε=0.2
ε=0.0
c^2
θ

Supplementary Figure 4 - Clustering coefficients at a fixed false positive rate

In order to analyze the combined effects of both false negative and false positive interactions the false positive rate (fraction of wrong interactions among all detected interactions) was set to a fixed value of 50%. Then, θ was chosen such that for each combination of β and ε approximately this false positive rate was obtained. This figure illustrates the resulting clustering coefficients at this fixed false positive rate for all network topologies and average degree values. The x coordinate represents edge coverage and the y coordinate bait the resulting average clustering coefficients. The different curves in each sub-figure show the results for different values of bait coverage.

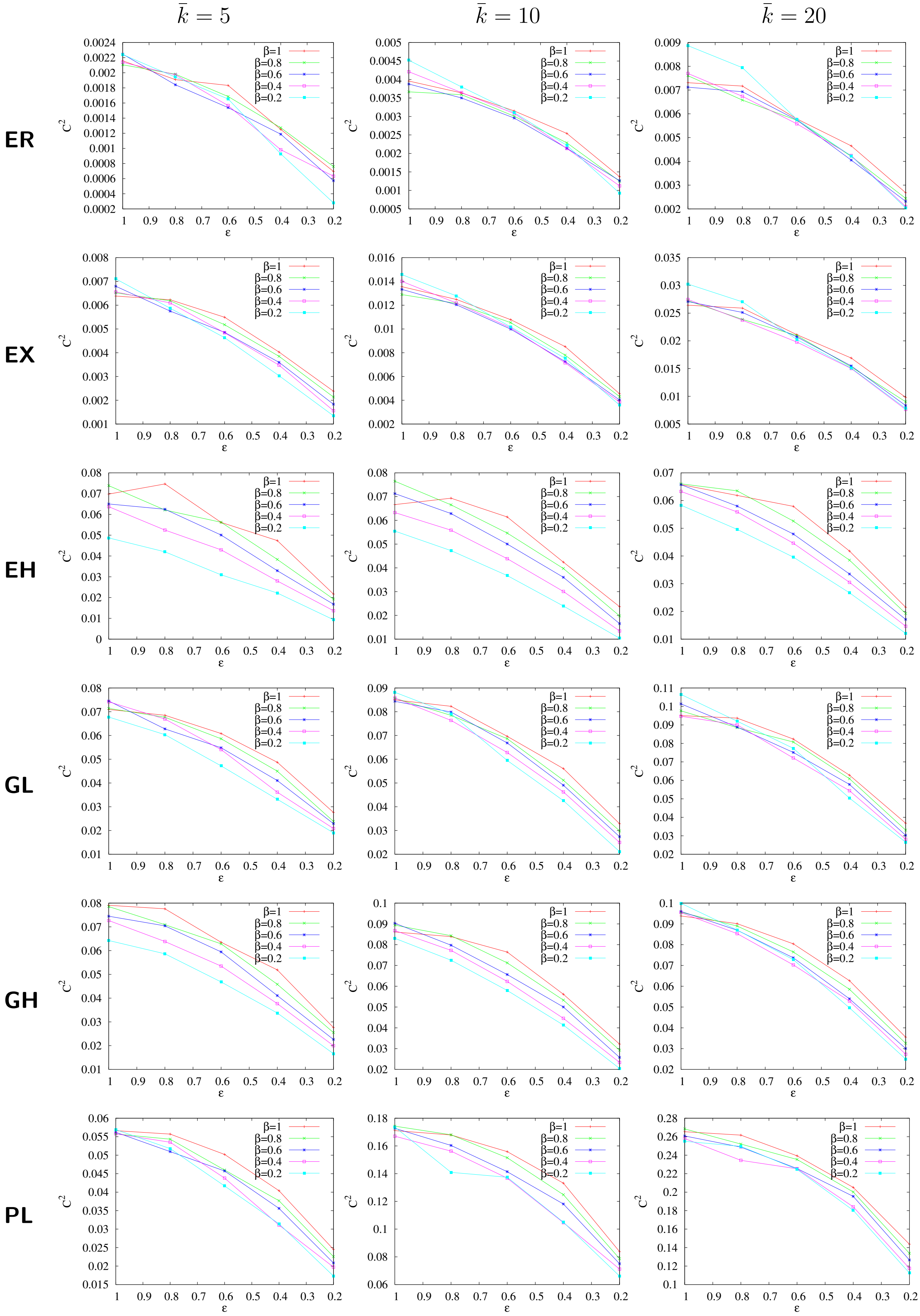
$\bar{k} = 5$
$\bar{k} = 10$
$\bar{k} = 20$
ER
EX
EH
GL
GH
PL
c^2
ε
β=1
β=0.8
β=0.6
β=0.4
β=0.2

References

[1] Erdős P, Rényi A: **On random graphs**. *Publicationes Mathematicae* 1959, **6**:290–297.

[2] Goffeau A, et al.: **Life with 6000 genes.** *Science* 1996, **274**(5287):546, 563–7.

[3] Friedel CC, Zimmer R: **Toward the complete interactome.** *Nat Biotechnol* 2006, **24**(6):614–615.
