# Supplementary material for: Inferring topology from clustering coefficients in protein-protein interaction networks
Source: BMC Bioinformatics. 2006 Nov 30;7:519. doi: 10.1186/1471-2105-7-519 (PMC1716184; doi:10.1186/1471-2105-7-519)
Supplement: Additional File 2 — Supplementary Methods. This file contains a description of the methods used for generating networks with different topologies. [file 1471-2105-7-519-S2.pdf]

Supplementary Methods
Generation of topology models

Networks of the ER, EX, GL and GH topologies were generated in the same way as described in [1]. For the sake of completeness, we again describe shortly the corresponding methods and the adaptations for the EH and PL networks.

Random graphs

Random graphs [2] (ER) are created using the following simple model [3]. In a network containing n nodes, each pair of nodes is connected with probability p. The distribution of node degrees in random graphs follows a Poisson distribution and the expected clustering coefficients is p.

Exponential and power-law networks

Exponential networks (EX and EH) are characterized by an exponential distribution of degree values such that $P(k) = \lambda e^{-\lambda k}$ for some constant λ. In power-law networks (PL) the probability of a node having degree k is proportional to $k^{-\gamma}$ for some constant γ. For our purposes, random networks with a given degree distribution are generated using the method described by Chung and Lu [4]. First an expected degree sequence $\mathbf{w} = (w_1, \ldots, w_n)$ is given for the n nodes which follows the chosen distribution. Then an edge is created between nodes v_i and v_j with probability $p_{ij} = w_i w_j \rho$ with $\rho = (1/\sum_i w_i)$. The w_i for the power-law networks are generated with an exponential cut-off as described by Newman *et al.* [5] using a two-step transformation/rejection method.

High clustering coefficients in exponential networks (EH) are created by introducing triangles into the network. Triangles are created by iteratively choosing a random node and connecting two random neighbors of this node. This is repeated until approximately the same clustering coefficients are obtained as for the GH networks. For small degree values, this leads to a deviation from the exponential distribution, but for the clustering coefficients considered, the deviation was only minor.

Growth model

To simulate the evolution of real protein interaction networks the growth model described by Vázquez *et al.* [6] is used. This model generates highly skewed networks (GL and GH), which are similar to power-law networks. For this purpose, starting from a small random graph, the following steps are repeated until the network contains the desired number of nodes n. First a node v is selected randomly and a new node v' is

created such that for each edge (v, u) we add an edge (v', u) with probability $1 - q$. The clustering coefficients of networks thus generated (GL) are lower than expected randomly, since many nodes which are connected to the same third node are often duplication pairs which are never connected.

Growth networks with high clustering coefficients (GH) are created by connecting v and v' through an additional edge with probability p. By increasing p the clustering coefficient of the network can be raised. However, there are limitations to the clustering coefficients which can be achieved with this approach without drastically changing the topology at small degree values.

References

1. Friedel CC, Zimmer R: **Toward the complete interactome.** *Nat Biotechnol* 2006, **24**(6):614–615.
2. Erdős P, Rényi A: **On random graphs**. *Publicationes Mathematicae* 1959, **6**:290–297.
3. Bollobás B: *Random Graphs*. Cambridge University Press 2001.
4. Chung F, Lu L: **The average distances in random graphs with given expected degrees.** *Proc Natl Acad Sci USA* 2002, **99**(25):15879–82.
5. Newman MEJ, Strogatz SH, Watts DJ: **Random graphs with arbitrary degree distributions and their applications**. *Phys. Rev. E* 2001, **64**(2):026118.
6. Vázquez A, Flammini A, Maritan A, Vespignani A: **Modeling of protein interaction networks**. *ComPlexUs* 2003, **1**:38–44.
